# Comprehensive Metabolomic Profiling and Biological Activity Analysis of *Eschscholzia californica* Extracts Using LC‐ESI‐QTOF‐MS

**DOI:** 10.1002/fsn3.70885

**Published:** 2025-09-02

**Authors:** Alina Kalyniukova, Shakeel Ahmed, Gunes Ak, Enver Saka, Tugce Duran, Nurgul Abul, Ilhami Gulcin, Ismail Senkardes, Mehmet Veysi Cetiz, Gokhan Zengin

**Affiliations:** ^1^ Faculty of Forestry and Wood Sciences Czech University of Life Sciences Prague Suchdol Czech Republic; ^2^ Physiology and Biochemistry Laboratory, Department of Biology, Science Faculty Selcuk University Konya Turkey; ^3^ Department of Medical Genetics, Faculty of Medicine KTO Karatay University Konya Turkey; ^4^ Department of Chemistry, Faculty of Sciences Ataturk University Erzurum Turkey; ^5^ Department of Pharmaceutical Botany, Faculty of Pharmacy Marmara University Istanbul Turkey; ^6^ Department of Medical Biochemistry, Faculty of Medicine Harran University Sanliurfa Turkey

**Keywords:** antioxidant capacity, enzyme inhibitory activity, *Eschscholzia californica*, in silico studies, LC‐ESI‐QTOF‐MS

## Abstract

The current investigation was designed to explore the chemical composition, antioxidant capacity, enzyme inhibitory activity, and cytotoxic potential of four different extracts (Ethyl Acetate, Ethanol, Ethanol/Water (70%) and Water) derived from the aerial parts of *Eschscholzia californica* . In vitro, assessments were performed utilizing diverse antioxidant assays, along with evaluations of neuroprotective enzyme inhibition targeting acetylcholine and butyl choline enzymes, as well as antidiabetic activities against α‐amylase and α‐glucosidase and a potential candidate for a tyrosinase inhibitor. LC‐ESI‐QTOF‐MS identification provided a total of 70 compounds in the extracted samples of *E. californica* , including kaempferol 3‐(deoxyhexosyl‐hexoside)‐7‐hexoside, rutin, quercetin dideoxyhexoside, caffeic acid hexoside, quinoline alkaloids, morphine derivatives, and scoulerine. Moreover, the ethanol extract exhibited the highest anti‐AChE (2.39 mg GALAE/g), while ethyl acetate exhibited anti‐BChE (3.31 mg GALAE/g), Ethanol/Water (70%) anti‐tyrosinase (53.09 mg KAE/g) and anti‐glucosidase (1.09 mmol ACAE/g) activities. Additionally, the ethyl acetate extract effectively inhibited carbonic anhydrase I and II isoenzymes. Furthermore, the ethanol and ethanol/water extracts demonstrated significant cytotoxicity against A549 lung cancer cells. However, *E. californica* partially/weakly triggers the apoptosis of cancer cells. Furthermore, the investigation identified 885 target genes for *E. californica* 's phytochemicals, 31 of which were familiar to insomnia. In silico studies demonstrated that protopine, rutin, eschscholtzidine, boldine, and (S)‐scoulerine exhibited notable inhibitory effects on insomnia‐related DRD5, DRD4, and SERT proteins. These findings highlight the potential pharmacological applications of the aerial parts of *E. californica* as a source for developing novel phytopharmaceuticals targeting various oxidative stress‐related conditions, including diabetes, cancer, Alzheimer's disease, and insomnia.

## Graphical abstract

The results indicate the potential pharmacological applications of *Eschscholzia californica* 's aerial parts as a source for the development of novel phytopharmaceuticals targeting conditions such as insomnia, schizophrenia, and depression.

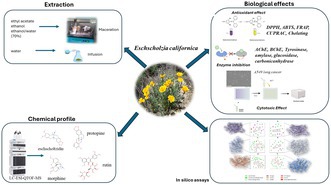

## 1 Introduction

The subject of herbal medicine has experienced a notable upsurge in the past few decades, drawing interest from developed and developing countries. Although not formally acknowledged by numerous nations, traditional medicine is extensively utilized in a substantial part of the globe. More than 80% of African and Asian populations predominantly depend on plants and plant extracts for health (Builders PF 2018). Many modern pharmaceutical drugs are sourced from natural compounds (De Luca et al. 2012). Studies in ethnopharmacology have gained global recognition as a valuable approach to discovering species that possess molecules or products with beneficial effects for pharmaceuticals, dietary supplements, and cosmetics (Harvey et al. 2015). In Western countries, there has been a significant rise in the use of medicinal plants. This fact can be attributed to growing concerns about the potential side effects of chemical drugs and the considerable financial benefits associated with natural remedies. It cannot be easy to accurately measure the economic benefits of the trade involving plants and plant extracts. However, industries related to traditional medicine are seeing significant annual growth rates of over 4% (Royal Botanic Gardens K 2016). Plants are a rich source of secondary metabolites used in food additives, agrochemicals, flavors, perfumes, colors, and biopesticides (Al‐Snafi 2017a). The diversity of beneficial bioactive molecules in medicinal species, including non‐conventional plants, is significant. These include phenolic compounds, carotenoids, tocopherols, and vitamins (Tlili and Sarikurkcu 2020).

Various diseases, including diabetes, cardiovascular disorders, cancer, and neurological ailments, are accelerated by reactive oxygen species (ROS), which are produced by a multitude of cellular metabolic pathways and environmental pollutants. According to numerous studies, plants and foods high in polyphenols have powerful antioxidant capabilities, which can scavenge free radicals and prevent diseases (Büyükbalci and El 2008). Prevention of vascular problems in people with diabetes is greatly assisted by antioxidants contained in plants, herbs, and nutritional sources (Aslan et al. 2010). Certain compounds in biology have antioxidant properties that react slowly. The way these biomolecules interact with each other in a mixture can affect their intended effects. When studying them, it is essential to consider their concentration and interactions (Pulido et al. 2000). As a result, there is a growing interest in investigating natural compounds that have significant biological effects and can be found in different plants and foods. Recent studies have brought attention to the inhibitory effects of these bioactive molecules on digestive enzymes like α‐amylase and α‐glucosidase, showcasing their potential in managing obesity and type 2 diabetes (Mopuri et al. 2018). As a result, a potential approach to thoroughly controlling diabetes is to use plants that have significant antioxidant and enzyme inhibitory effects.

The genus *Eschscholzia* is represented worldwide with 10 to 12 species (Clark 1997). The California poppy, scientifically known as *Eschscholzia californica* Cham, belongs to the Papaveraceae family. It is indigenous to southern California and northern Mexico and has traits of both permanent and annual plants. *E. californica* is globally used due to its well‐known sedative, anxiolytic, analgesic, and antinociceptive effects (Rolland et al. 2001; Schäfer et al. 1995; Hanus et al. 2004). The plant contains a variety of medically active substances, mainly alkaloids such as chelirubine, californidine, sanguinarine, and macarpine, as well as flavonoids, specifically quercetin, isorhamnetin, and glycosides, which are primarily found in its above‐ground sections (Al‐Snafi 2017b). The species is not native to Turkey and has invasive characteristics (Asal et al. 2022). As far as prior studies are concerned, none have examined the effects of different extraction solvents on the chemical makeup, antioxidant potential, and enzyme inhibition potential of *E. californica* . As a result, this work is the first to comprehensively evaluate the antioxidant potential of several extracts made from *E. californica* using a variety of solvents. Additionally, the study broadens its focus by assessing these extracts' ability to inhibit enzymes, which adds a critical component to our understanding of their bioactive properties.

## 2 Materials and Methods

### 2.1 Plant Collection

Plant materials (in their flowering phase) were obtained from the Kartal location (Dragos), Istanbul, Turkey in 2021. Dr. A voucher specimen with voucher number MARE‐22674 was stored in Marmara University's herbarium after Ismail Senkardes conducted taxonomic identification. Carefully separating the aerial parts, drying them in the shade at room temperature, grinding them, and storing them in darkness was done.

### 2.2 Plant Extract Preparation

To produce extracts, four solvents, including ethyl acetate, ethanol, ethanol/water (70%), and water, were employed. For 24 h at room temperature, the sample was mixed with 200 mL of ethanol, ethanol, and ethanol/water to macerate it. The infusion method was employed to extract the water from 10 g of plant material after it was soaked for 15 min with boiled water. The process involved evaporating the organic solvents under reduced pressure and lyophilizing the water extract with a freeze dryer.

### 2.3 Assay for Total Phenolic and Flavonoid Contents

The measurement of total phenolics and flavonoids was carried out based on the procedures detailed by (Slinkard and Singleton 1977); total phenolics and flavonoids were measured. Gallic acid (GA) and rutin (RE) provided reference in the experiments, with the results displayed as gallic acid equivalents (GAE) and rutin equivalents (RE).

### 2.4 LC‐ESI‐QTOF‐MS Metabolomic Analysis

LC‐ESI‐QTOF‐MS metabolomic analysis was performed using an Agilent 1290 Infinity II system coupled with an Agilent 6546 LC/MS QTOF instrument (Agilent, USA). A column of InfinityLab Poroshell 120 EC‐C18 (2 × 150 mm, 2.7 μm) was used to separate compounds from Agilent (USA). Other analytical details are given in the Supporting Information.

### 2.5 Assays for In Vitro Antioxidant Capacity

Utilizing the procedures specified by (Grochowski et al. 2017), tests for antioxidants were carried out. Measurements obtained from the DPPH, ABTS radical scavenging, CUPRAC, and FRAP evaluations were reported in milligrams of Trolox equivalents (TE) per gram of the extract. The antioxidant capacity was measured in millimoles of Trolox equivalents (TE) per gram of extract, as demonstrated by the phosphomolybdenum (PBD) assay. The activity of metal chelation (MCA) was quantified in terms of milligrams of disodium edetate equivalents (EDTAE) for each gram of the extract.

### 2.6 Inhibitory Effects Against Some Key Enzymes

Following standard procedures (Grochowski et al. 2017), experiments to inhibit enzymes were carried out on the samples. Acarbose equivalents (ACAE) per gram of extract were used to quantify the inhibition of amylase and β‐glucosidase. On the other hand, the suppression of acetylcholinesterase (AChE) and butyrylcholinesterase (BChE) was expressed in milligrams of galanthamine equivalents (GALAE) per gram of the extract. The assessment of tyrosinase inhibition involved determining the quantity of kojic acid equivalents (KAE) in milligrams per gram of the extract. In the experiments, the carbonic anhydrase isoenzymes (hCA I‐II) were extracted from human erythrocytes by employing Sepharose‐4B‐L‐Tyrosine sulfanilamide affinity chromatography, as outlined in earlier research (Kucukoglu et al. 2019; Özbey et al. 2016).

### 2.7 Cell Culture

The A549 non‐small‐cell lung cancer cells and HEK293 human embryonic kidney cells were obtained from ATCC (American Type Culture Collection; Rockville, MD, USA). The cells were monolayer cultured in DMEM fresh medium supplemented with 10% heat‐activated fetal bovine serum (FBS; Biochrome, Germany) and 1% penicillin/streptomycin (100 U/mL penicillin and 100 μg/mL streptomycin) antibiotic solution (Sigma‐Aldrich) at 37°C in a humidified atmosphere of 5% CO_2_. When cells reached ~70% to 90% confluency, they were passaged with 0.05% w/v trypsin–EDTA after washing with 1xPBS (Gibco, USA) and cryopreserved at −80°C in a 10% DMSO medium.

### 2.8 MTT Cytotoxicity Test

After detachment with trypsin–EDTA, the cells were de‐trypsinized with a fresh medium. Immediately afterward, they were centrifuged at 1500 rpm for 5 min. The cell pellet was suspended in fresh DMEM medium, and equal numbers of cells (~5 × 10^3^ cells/well) were cultured in 96‐well plate wells. After ~24 h, the medium was removed, and different doses of extracts (10, 50, 100, 250, 500, 750, 1000 μg/mL) were added to the cells. At the end of the 24‐, 48‐, and 72‐h periods, MTT dye was added, and the plates were incubated at 37°C for 4 h. Formazan crystals were dissolved in DMSO, and the plates were incubated in the dark for 5 min at room temperature. Absorbance measurement was taken at 570 nm in a microplate reader (Multiskan Go, Thermo Scientific, USA). The IC50 value was calculated with the following formula: Cell viability (%) = A570 nm of treated cells/A570 nm of control cells x 100 (Duran and Tuncer 2023).

### 2.9 RNA Isolation and cDNA Synthesis

For total RNA isolation, cells for which the IC_50_ value was applied and cells for which the IC_50_ value was not used were unattached separately with trypsin–EDTA. The cell pellet was gently treated with TRIzol reagent (Invitrogen, Thermo Fisher Scientific, Waltham, USA). After the resulting suspension was treated with fresh chloroform and isoamyl alcohol, total RNA was precipitated at 13,000 × g for 25 min. After the alcohol was evaporated at room temperature, the RNA pellet was dissolved in an appropriate volume of nuclease‐free water. After the concentrations of all RNA samples were equalized (0.1 ng‐5 μg), cDNA synthesis was performed with the RevertAid First Strand cDNA Synthesis Kit (Thermo Scientific, USA).

### 2.10 Real‐Time PCR and Statistical Analysis

Expression levels of apoptosis‐related genes (Table S2) were investigated using the QuantStudio 3 Real‐Time PCR system (Thermo Fisher Scientific). SYBR Green PCR Mix, cDNA sample, and specific primers were gently pipetted into the MicroAmp Fast Optical 96‐Well Reaction Plate, with a total volume of 10 μL. After the plate was covered with ABSolute qPCR Plate Seal (Thermo Fisher Scientific) in an airtight manner, 40 cycles of qPCR reaction were set up: 95°C, 5 min denaturation, and 30 s amplification at 57°C to 60°C data was recorded. After normalizing with *GAPDH*'s Ct value, treated (experimental)/untreated (control) ∆Ct values were analyzed using Livak's ∆∆Ct method (Livak and Schmittgen 2001). A value of *p* < 0.05 was considered statistically significant.

### 2.11 Screening of Bioactive Compound‐Associated Protein Targets

Determining therapeutic targets constitutes a fundamental stage in the drug discovery process within biomedical research. In this regard, resources such as STRING, DisGeNET, the Comparative Toxicogenomics Database (CTD), and GeneCards serve as integral platforms for the identification of candidate drug targets, particularly in oncological studies. A search for the term “insomnia” in these four databases revealed insomnia‐related genes. Genes associated with the bioactive compounds were systematically extracted from multiple databases, including SwissTarget, STITCH, CTD, and PubChem. To elucidate the potential mechanisms of action of these compounds in the context of insomnia, shared molecular targets were identified through comparative analysis using the Venny v2.1.0 tool (Akpulat et al. 2025; Hryć et al. 2025).

### 2.12 Protein–Protein Interaction (PPI) Network Analysis

A protein–protein interaction (PPI) network was developed using STRING v12.0 to evaluate the anti‐insomnia potential of *Eschscholzia californica* . The analysis prioritized biologically meaningful associations between proteins, applying a confidence score threshold of 0.4 to ensure robustness. The network was limited to *Homo sapiens* , and visualization of the resulting interactions was carried out via Cytoscape v3.10.2. To identify core targets within the network, the CytoHubba plugin was used to highlight key proteins potentially involved in the anti‐insomnia mechanisms of *E. californica* . This approach facilitated the inference of a regulatory network underlying the observed pharmacological effects (Cetiz et al. 2024; Llorent‐Martínez et al. 2025).

### 2.13 Functional Annotation and Pathway Enrichment via GO and KEGG Frameworks

A series of functional enrichment analyses was carried out using the DAVID v6.8 bioinformatics platform, incorporating Gene Ontology (GO) terms and KEGG pathway annotations. GO terms were stratified into three primary categories: molecular function (MF), cellular component (CC), and biological process (BP). Enrichment results with *p*‐values less than 0.05 were considered statistically significant. Visualization of these outcomes was performed using a bioinformatics resource (http://www.bioinformatics.com.cn/) (Bahsi et al. 2025; Cetiz, Ahmed, et al. 2025; Cetiz, Yagi, et al. 2025; Zheleva‐Dimitrova et al. 2025).

### 2.14 Molecular Docking

Protein 3D structures were sourced from the Protein Data Bank (PDB), while ligand molecules were retrieved from the PubChem database. Water molecules and cofactors were eliminated using BIOVIA Discovery Studio Visualizer v4.5. Ligand structures were initially converted using OpenBabel v3.1.1 and subsequently subjected to energy minimization in Avogadro v0.8.0 employing the MMFF94 force field. Preparation of both proteins and ligands—inclusive of polar hydrogen incorporation and charge calculations—was carried out via MGLTools v1.5.6. Binding site identification was conducted using POCASA v1.1, and visual validation was performed in PyMOL v2.5.8 (Hetmann et al. 2023; Yagi et al. 2024). Molecular docking simulations were executed with AutoDock Vina v1.1.2, using predefined grid box parameters as described by Trott and Olson (Trott and Olson 2010).

### 2.15 Adme/T Analysis

The canonical SMILES structures of 18 *E. californica* compounds were retrieved from PubChem. Their ADMET profiles were predicted. Their ADMET properties were estimated through the pkCSM web‐based platform (https://biosig.lab.uq.edu.au/pkcsm/). The evaluation encompassed aqueous solubility, gastrointestinal uptake, and penetration across the blood–brain barrier (BBB), as well as fraction unbound and potential hepatotoxicity (Pires et al. 2015).

## 3 Results and Discussion

### 3.1 Total Phenolic and Flavonoid Content

Phenolic compounds, essential constituents of plants, exhibit redox properties vital to their antioxidant functionality (Soobrattee et al. 2005; Nilofar, Dall'Acqua, et al. 2024). The hydroxyl groups present in plant extracts are significant in facilitating the elimination of free radicals. Table 1 elucidates the quantitative analysis of Total Phenolic Content (TPC) and Total Flavonoid Content (TFC) within various extracts of *E. californica* . The TPC, expressed in milligrams of Gallic Acid Equivalent per gram (mg GAE/g), indicates the concentration of phenolic compounds. In contrast, the TFC, measured in milligrams of Rutin Equivalent per gram (mg RE/g), provides insight into the flavonoid composition. The Ethanol/Water (70%) extract exhibits the highest TPC value, showcasing a robust phenolic profile at 32.99 mg GAE/g. Conversely, the Ethyl acetate extract demonstrates the lowest TPC, indicating a comparatively lower abundance of phenolic compounds at 23.78 mg GAE/g.

**TABLE 1:** Total phenolic and flavonoid contents in the tested extracts*.

Extracts	TPC (mg GAE/g)	TFC (mg RE/g)
Ethyl acetate	23.78 ± 0.51^c^	5.83 ± 0.31^d^
Ethanol	32.73 ± 0.74^a^	18.85 ± 0.35^c^
Ethanol/Water (70%)	32.99 ± 0.17^a^	27.38 ± 1.00^a^
Water (infused)	29.17 ± 0.54^b^	21.52 ± 0.06^b^

*Note:* * Values are reported as mean ± SD of three parallel measurements. Different letters indicate the differences between the extracts (*p* < 0.05).

Abbreviations: GAE, gallic acid equivalents; RE, rutin equivalents.

Moreover, regarding TFC, the Ethanol extract emerges as the frontrunner, boasting a notable flavonoid content of 18.85 mg RE/g. In stark contrast, the Ethyl acetate extract records the lowest TFC, suggesting a relatively modest flavonoid presence at 5.83 mg RE/g. These findings not only shed light on the diverse phenolic and flavonoid compositions among the extracts but also underscore the influence of extraction solvents on the bioactive compound yield, thereby offering valuable insights for potential applications in various fields such as pharmaceuticals, nutraceuticals, and food science.

### 3.2 Chemical Characterization

**FIGURE 1:**
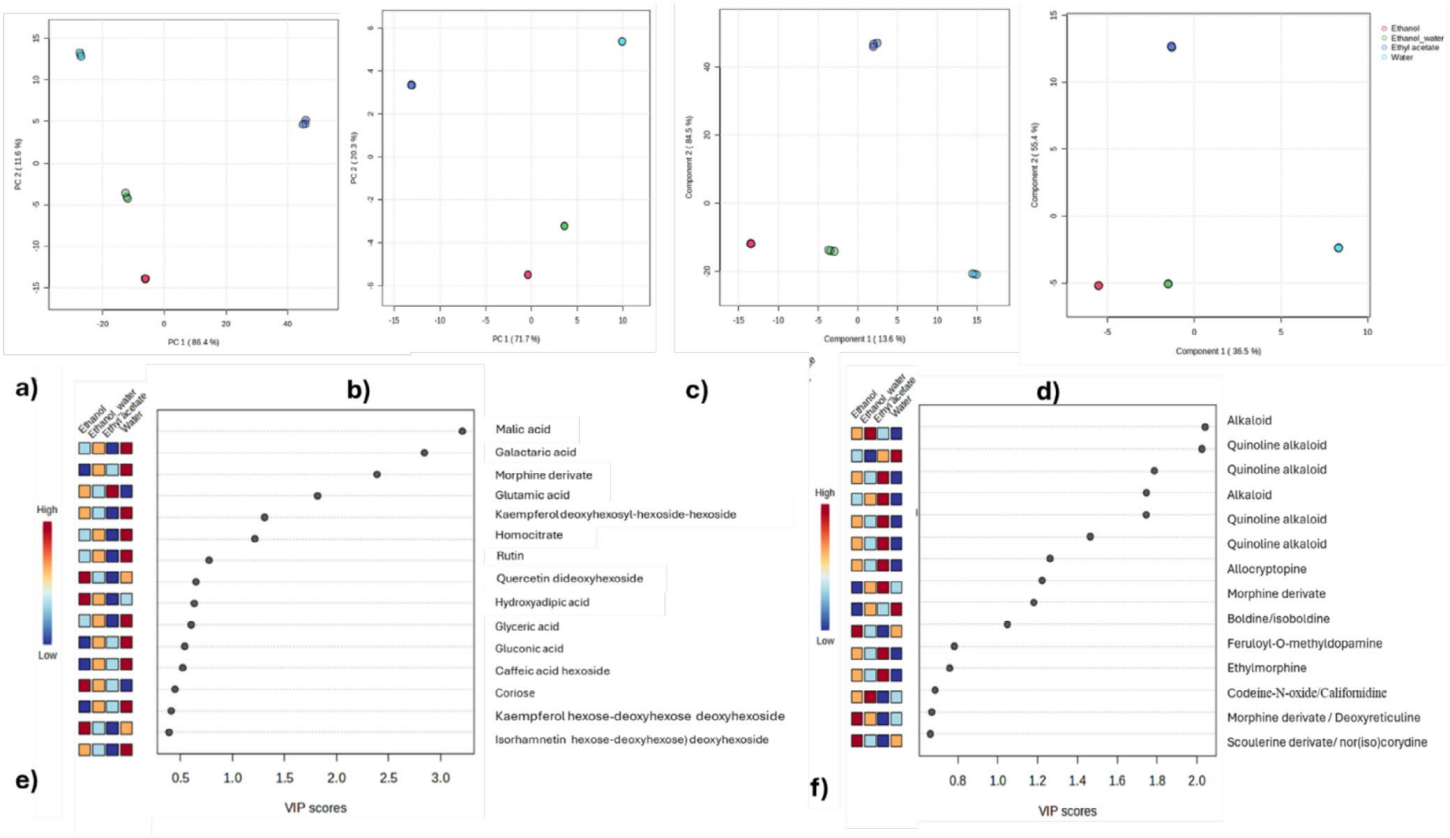
PCA and PLSDA scores plot. PCA derived (a) from negative and (b) from positive ionization modes. PLSDA score plots (c) from negative and (d) from positive ionization modes. PLSDA loadings for the 15 most important variables in (e) negative and (f) positive ionization mode.

**FIGURE 2:**
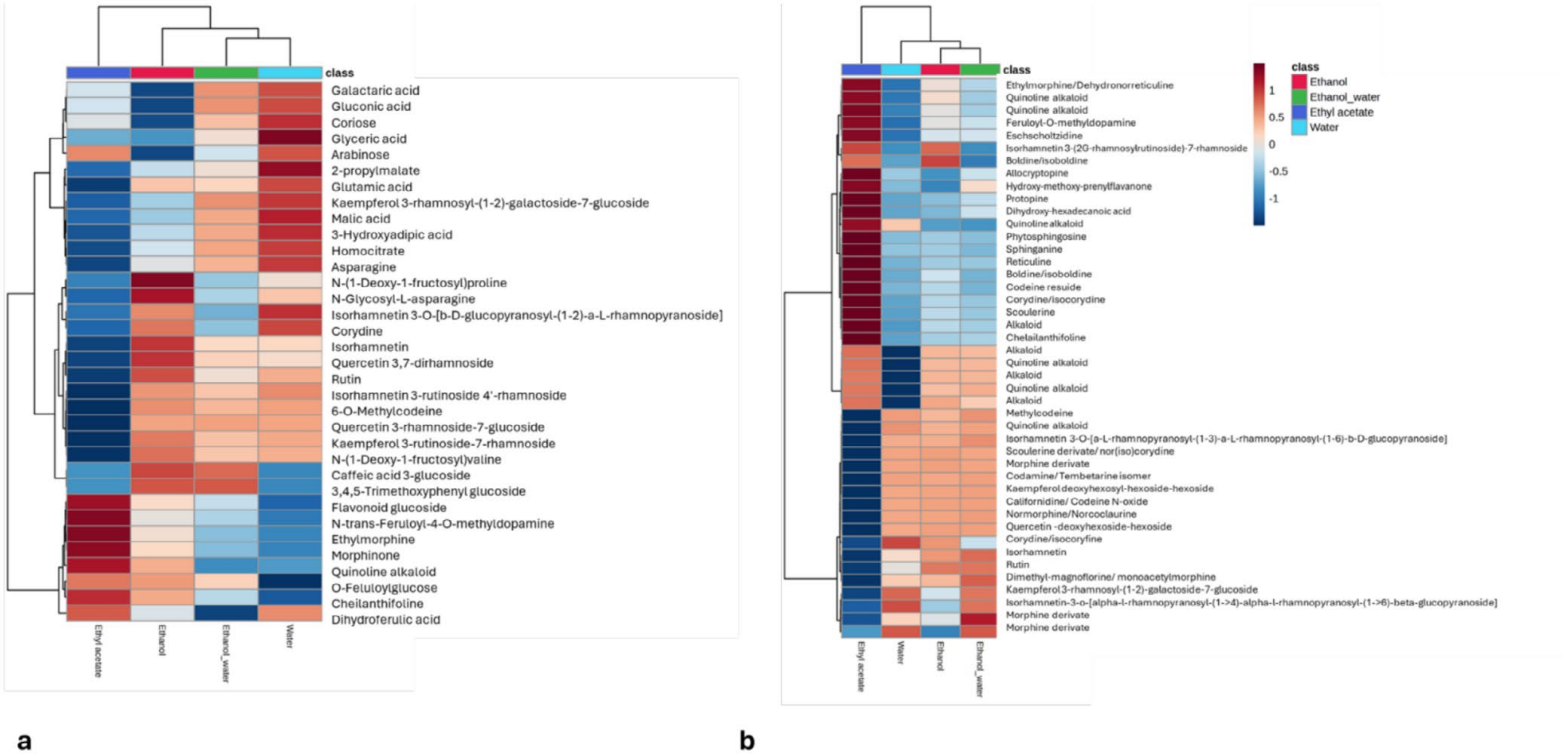
Heatmap of metabolites in four extracts, sorted via ANOVA test (*p* ≤ 0.05). (a) Negative and (b) positive ionization mode: row: samples; columns: metabolites.

Metabolomic analysis was performed to characterize plant extracts chemically. Herein, we applied Principal Component Analysis (PCA) and Partial Least Squares Discriminant Analysis (PLSDA) to investigate four extracts of *E. californica* in both positive and negative ionization modes to elucidate chemical diversity and differences among plant extracts. 138 and 288 features were extracted in negative and positive ionization modes, resp., while 65 compounds were identified. The list of identified compounds is presented in Table S1, and their abundance in the samples is shown in Tables S1 and S2. From Figure 1a,b, it is seen that PC1 occupied 86.4% and PC2 11.6% of the total variance for the negative ionization mode. In comparison, PC1 occupied 71.7% and PC2 20.3% of the total variance for the positive ionization mode, showing apparent differences in plant extract. Using the supervised PLS‐DA approach (Figure 1c,d), the analysis focused on the 15 most significant variables, selected based on a VIP score of ≥ 1 in the PLS‐DA model to enhance differentiation among the four groups. This subset of variables demonstrated clear separation across the groups. Notably, in the negative ionization mode, variations in extraction efficiency within plant extracts were observed for various amino acids such as malic acid, galactaric acid, glutamic acid, and gluconic acid; polyphenolic compounds including kaempferol 3‐(deoxyhexosyl‐hexoside)‐7‐hexoside, rutin, quercetin dideoxyhexoside, caffeic acid hexoside, kaempferol‐(deoxyhexosyl‐hexoside)‐hexoside, isorhamnetin‐3‐o‐[alpha‐l‐rhamnopyranosyl‐(1‐ > 4)‐alpha‐l‐rhamnopyranosyl‐(1‐ > 6)‐beta‐glucopyranoside], and a derivative of morphine were significant features for group differentiation. Early studies revealed that flavonoids in *Eschscholzia californica* are predominantly present as glycosides of quercetin and isorhamnetin (Beck and Häberlein 1999). Furthermore, in the positive ionization mode, distinct variations in extraction efficiency were primarily observed in quinoline alkaloids with m/z 355, alkaloid isomers with m/z 341, morphine derivatives with m/z 313, scoulerine derivatives with m/z 327, codeine residue, codamine or tembetarine isomer, as well as polyphenolic compounds like those in the negative ionization mode. Additionally, hierarchical clustering heatmapping provided (Figure 2) a relatively quantitative description of metabolite profiles depending on their abundance, sorted via ANOVA test (*p* ≤ 0.05), emphasizing solvent‐dependent variations in metabolite concentrations. Ethyl acetate extracts exhibited higher concentrations of quinoline alkaloids, whereas polyphenolic compounds were more efficiently extracted using ethanol and Ethanol/Water (70%) solvents, and amino acids were enriched in water (infused) extracts. Also, the alkaloids predominantly identified in *Eschscholzia californica* belong to several distinct classes, including protopines (e.g., protopine and allocryptopine), aporphine alkaloids (e.g., magnoflorine, corydine, and isoboldine), simple benzylisoquinolines (e.g., reticuline), and pavine alkaloids (e.g., californidine and escholtzine) (Becker et al. 2023).

### 3.3 Antioxidant Effects

A wide range of assays can be used to directly measure the transfer of hydrogen atoms or electrons from potential antioxidants to free radicals in simplified “lipid‐free” systems. The antioxidant activities obtained through these methods are often linked to their ability to eliminate certain types of radical species, some of which may be artificially generated and not biologically relevant (Shahidi and Zhong 2015; Elhawary et al. 2024). Many different antioxidant assays are used to assess the antioxidant potential of natural products. This study used six different antioxidant assays, including DPPH, ABTS, CUPRAC, FRAP, PBD, and MCA. The Ethanol/Water (70%) extract demonstrates the highest antioxidant activity in various tests, suggesting its strong ability to scavenge free radicals. In the DPPH experiment, this extract has a significant scavenging activity, surpassing all other extracts, with a value of 52.06 mg TE/g.

Similarly, the ABTS assay shows a remarkable antioxidant capacity of 105.73 mg TE/g. In addition, the Ethanol/Water (70%) extract consistently demonstrates higher antioxidant activity than the other extracts in the CUPRAC and FRAP assays, with values of 90.88 and 70.25 mg TE/g, respectively. The ethyl acetate extract showed the lowest values for most of the evaluated methods except for PBD, where it provides the highest activity among all the tested extracts with the value of 2.09 mmol TE/g, followed by ethanol, Ethanol/Water (70%) and water (infused) extracts, respectively (Table 2).

**TABLE 2:** Antioxidant properties of the tested extracts*.

Extracts	DPPH (mg TE/g)	ABTS (mg TE/g)	CUPRAC (mg TE/g)	FRAP (mg TE/g)	Chelating (mg EDTAE/g)	PBD (mmol TE/g)
Ethyl acetate	14.18 ± 0.12^d^	46.32 ± 3.80^d^	52.52 ± 0.75^c^	27.84 ± 0.79^d^	1.37 ± 0.25^c^	2.09 ± 0.09^a^
Ethanol	39.28 ± 0.83^c^	81.98 ± 1.18^c^	88.33 ± 1.10^a^	63.59 ± 0.57^b^	7.14 ± 0.58^b^	0.91 ± 0.02^b^
Ethanol/Water (70%)	52.06 ± 0.42^a^	105.73 ± 1.10^a^	90.88 ± 1.26^a^	70.25 ± 1.42^a^	21.21 ± 0.41^a^	0.72 ± 0.03^c^
Water (infused)	45.26 ± 0.70^b^	92.89 ± 1.82^b^	58.51 ± 1.65^b^	55.52 ± 0.84^c^	20.70 ± 0.19^a^	0.61 ± 0.04^c^

*Note:* * Values are reported as mean ± SD of three parallel measurements. Different letters indicate the differences between the extracts (*p* < 0.05).

Abbreviations: EDTAE, EDTA equivalent; PBD, Phosphomolybdenum; MCA, Metal chelating activity; TE, Trolox equivalent.

A correlation can be detected when comparing antioxidant activity with the previously discussed Total Phenolic Content (TPC) and Total Flavonoid Content (TFC). Extracts with higher total phenolic content (TPC) and total flavonoid content (TFC) typically have more antioxidant activity. This is because phenolic and flavonoid components are recognized for their robust antioxidant characteristics (Nilofar, Dall'Acqua, et al. 2024). For example, the Ethanol/Water (70%) extract, which showed the greatest Total Phenolic Content (TPC) and Total Flavonoid Content (TFC) values, also exhibits the most potent antioxidant activity in most tests. Overall, our results from the six in vitro assays suggested that extracts' antioxidant capacities strongly rely on the solvent and quantities of phenolic and flavonoid contents in the extracts. This may be associated with the chemical structure of phenolic compounds and the availability of phenolic hydroxyl groups, which can donate their electron or hydrogen, forming stable end products (Kobus‐Cisowska et al. 2019).

On the other hand, extracts that have lower total phenolic content (TPC) and total flavonoid content (TFC), such as the Ethyl acetate extract, have somewhat reduced antioxidant activity. This link highlights the significance of phenolic and flavonoid components in determining the antioxidant capacity of plant extracts. The findings emphasize the notable antioxidant capability of the Ethanol/Water (70%) extract, indicating its potential usefulness in diverse applications targeting the treatment of oxidative stress‐related illnesses or the preservation of food and pharmaceutical items. In another study, Bonomo et al. found a weaker antioxidant potential for the *E. californica* (Bonomo et al. 2023). In another study, *Argemone mexicana* , a plant species from the same family, Papaveraceae, exhibited a strong antioxidant potential by DPPH and ABTS scavenging assays (Datkhile et al. 2020).

### 3.4 Enzyme Inhibitory Effects

An essential tool in drug discovery is the use of enzyme inhibitors. Many diseases are caused by malfunctioning, overexpressed, or hyperactivated enzymes, as demonstrated by developments in molecular biology. Enzyme overexpression or hyperactivation can be treated using enzyme inhibitors. Several enzyme inhibitors, some naturally occurring, are now being used in therapeutic settings because of these efforts (Zengin et al. 2023).

Different extracts of *E. californica* were used to investigate the potential for enzyme inhibition of several clinically relevant enzymes involved in significant pathologies, such as neurological disorders (AChE and BChE), skin diseases (tyrosinase), and diabetes (β‐glucosidase and α‐amylase). Table 3 provides a summary of the test results. The quantification of these activities is measured in different units for each enzyme: milligrams of Galantamine Equivalent per gram (mg GALAE/g) for AChE and BChE, milligrams of Kojic Acid Equivalent per gram (mg KAE/g) for tyrosinase, and millimoles of Acarbose Equivalent per gram (mmol ACAE/g) for α‐amylase and α‐glucosidase.

**TABLE 3:** Enzyme inhibitory properties of the tested extracts*.

Extracts	AChE (mg GALAE/g)	BChE (mg GALAE/g)	Tyrosinase (mg KAE/g)	Amylase (mmol ACAE/g)	Glucosidase (mmol ACAE/g)
Ethyl acetate	1.81 ± 0.19^b^	3.31 ± 0.37^a^	45.88 ± 4.88^b^	0.74 ± 0.01^a^	0.17 ± 0.01^b^
Ethanol	2.39 ± 0.02^a^	2.82 ± 0.35^ab^	50.70 ± 0.55^ab^	0.60 ± 0.01^b^	0.92 ± 0.11^a^
Ethanol/Water (70%)	2.33 ± 0.02^a^	1.44 ± 0.37^c^	53.09 ± 1.06^a^	0.46 ± 0.02^c^	1.09 ± 0.16^a^
Water (infused)	2.38 ± 0.05^a^	2.06 ± 0.08^bc^	na	0.08 ± 0.01^d^	0.34 ± 0.01^b^

*Note:* * Values are reported as mean ± SD of three parallel measurements. Different letters indicate the differences between the extracts (*p* < 0.05).

Abbreviations: ACAE, Acarbose equivalent; GALAE, Galantamine equivalent; KAE, Kojic acid equivalent; na, not active.

### 3.5 Cholinesterase Inhibition

Alzheimer's disease (AD), which affects an estimated 47 million people worldwide, is expected to become an increasingly significant socio‐economic issue in the years to come due to the aging population (Karch et al. 2018; Prince et al. 2015). Oxidative stress, triggered by the production of reactive oxygen species (ROS), is a significant factor in this phenomenon. One crucial therapeutic approach is to suppress the enzymes acetylcholinesterase (AChE) and butyrylcholinesterase (BChE), which have substantial but modest impacts on disease modification (Acquaviva et al. 2023). It has been acknowledged that phytochemicals are known for various biological characteristics, such as the inhibition of AChE and BChE (Torres et al. 2022). Furthermore, they demonstrate the potential for reducing ROS levels and interfering with inflammatory pathways, which could improve the management of AD (Ferreira et al. 2006; Marucci et al. 2021).

The potential of *E. californica* extracts in promoting nervous system health was investigated by evaluating their ability to inhibit AChE and BChE enzymes. The breakdown of acetylcholine by AChE is crucial for transmitting nerve impulses, which is vital for cognitive function and muscle contraction. In addition, BChE plays a role in the hydrolysis of choline esters, although it does not directly contribute to the transmission of nerve impulses. The study's findings, as presented in Table 3, demonstrate that the extracts' effectiveness in inhibiting AChE and BChE varied between 2.39 and 1.81 mg galantamine equivalent (GALAE)/g dry extract and 3.31 to 1.44 mg GALAE/g dry extract, respectively. The activity of AChE inhibition decreased in the following sequence: the order of activity for the first set of compounds was Ethanol>Water (infused) > Ethanol/Water (70%) > Ethyl acetate. On the other hand, for BChE, the order of activity was Ethyl acetate>Ethanol > Water (infused) > Ethanol/Water (70%). Based on the findings, the extract exhibited significant inhibition of the BChE enzyme compared to AChE activity, consistent with a prior investigation where the extracted compounds demonstrated notable efficacy against BChE rather than AChE enzyme inhibition (Cahlíková et al. 2010). Curiously, no apparent link was found with polyphenolic composition. Extracts with the highest polyphenolic content did not always show the strongest cholinesterase inhibition. Surprisingly, the extracts from Ethyl acetate showed the least affinity for inhibiting the AChE enzyme and gave the maximum inhibition for BChE. This highlights the role of various compounds with different structures and how they work together to inhibit these crucial enzymes related to neurodegenerative disorders.

### 3.6 α‐Amylase and α‐Glucosidase Inhibition

Type 2 diabetes presents a significant health challenge on a global scale, leading the World Health Organization (WHO) to support the exploration of alternative medicines for its treatment (Godman et al. 2020). The condition previously referred to as maturity‐onset diabetes has become more prevalent, even among children, due to the increasing rates of obesity (Twig et al. 2020). The main goal of treatment is to keep blood glucose levels within a normal range to prevent complications like skin infections, diabetic nephropathy, and cardiovascular disorders (Dwivedi and Pandey 2020). Regulating blood glucose levels can be achieved by inhibiting digestive enzymes such as α‐amylase and α‐glucosidase, which is considered a valuable approach. Natural products provide a promising option for intervention, thanks to their limited side effects and improved tolerability compared to traditional oral hypoglycemic agents (Papoutsis et al. 2021). This highlights the urgent requirement for successful therapeutic interventions, emphasizing the importance of studying natural products that may have anti‐diabetic properties. It is a crucial field of modern medical research. Considering this, our study sought to investigate the bioactive properties of different extracts of *E. californica* that are relevant for managing hyperglycemia and type 2 diabetes. The attributes described involve the inhibition of α‐amylase and α‐glucosidase enzymes.

The enzyme inhibition profiles of different extracts, specifically α‐amylase and α‐glucosidase, provide intriguing insights that may lead to the development of therapeutic applications. Significant inhibition activity of α‐amylase was observed in the ethyl acetate and ethanol extracts. The corresponding values for these extracts are 0.74 and 0.60 mmol ACAE/g, respectively (Table 3). In contrast, the water (infused) extract exhibits the lowest capacity for inhibiting at 0.08 mmol ACAE/g. The inhibitory effects of α‐glucosidase, which are crucial for regulating the metabolism of carbohydrates, are also observed in various ways across the extracts. The ethanol extract demonstrates the greatest inhibition activity at 0.92 mmol ACAE/g. However, the ethanol/water (70%) extract exhibits even higher inhibition, with respective values of 1.09 ± 0.16 mmol ACAE/g. On the contrary, the water (infused) and ethyl acetate extracts exhibit a comparatively diminished capacity to inhibit both enzymes, indicating the possibility of divergent mechanisms underlying their respective actions. The results of this study emphasize the wide range of enzyme inhibition profiles exhibited by the extracts.

### 3.7 Tyrosinase Inhibition

The application of botanical remedies as tyrosinase inhibitors is widespread in the pharmaceutical and cosmetic sectors. Tyrosinase is the principal enzyme in the biosynthesis of melanin, a phenomenon called melanogenesis. The hydroxylation of l‐tyrosine is initiated by tyrosinase, resulting in the formation of l‐dihydroxyphenylalanine (l‐DOPA). Following this, tyrosinase catalyzes the oxidation of l‐DOPA to produce dopaquinone, which subsequently initiates a sequence of reactions culminating in the formation of melanin (Gogoi et al. 2023). Although it protects against ultraviolet (UV) damage, melanin can cause a range of pathological conditions when produced in excess, such as lentigo, congenital melanocytic naevi, melasma, and others (Choi et al. 2022; Vaezi 2022). As a result, the regulation of tyrosinase activity becomes a potentially practical approach for managing melanin synthesis (Nilofar, Uba, et al. 2024).

The findings regarding the inhibitory effects of extracts of *E. californica* on the tyrosinase enzyme are displayed in Table 3. The results indicate that tyrosinase activity was detectable in all extracts except for water (infused). The tyrosinase activity values varied between 53.09 and 45.88 mg kojic acid equivalent (KAE)/g dried extract. Specifically, the tyrosinase activity was greatest in the ethanol/water (70%) extract (53.09 mg KAE/g), followed by the ethanol and ethyl acetate extracts (50.75 and 45.88 mg KAE/g dried extract, respectively). On the contrary, the infused water extract failed to demonstrate the inhibitory effect of tyrosinase activity.

Some correlations may also emerge when considering the antioxidant activity discussed earlier, especially in comparison to enzyme inhibition activity. Phenolic and flavonoid compounds, renowned for their antioxidant properties, can also affect enzyme inhibition activity by interacting with enzymes (Gonçalves and Romano 2017). Therefore, extracts with a higher level of antioxidant activity may also demonstrate more potent enzyme inhibition activity. However, more research is needed to establish direct connections between the antioxidant and enzyme inhibition activities found in these extracts. The findings indicate that the Ethanol/Water (70%) extract has notable bioactivity in different assays, suggesting its potential as a therapeutic option for conditions associated with enzyme dysregulation and oxidative stress.

### 3.8 Inhibition of Carbonic Anhydrase Isoenzymes I and II

Several investigations on carbonic anhydrases (CAs) have shown their substantial involvement in a range of disorders, such as epilepsy, high altitude sickness, glaucoma, sleep apnea, obesity, neuropathic pain, and sleep apnea (Scozzafava et al. 2004; Winum et al. 2006; Temperini and Scozzafava 2009). Therefore, numerous isoforms of human carbonic anhydrase (hCA) are important targets for developing inhibitors with practical uses in medicine. Carbonic anhydrase II (CA II) is widely present in several tissues and is associated with conditions such as epilepsy, glaucoma, high altitude sickness, edema, and renal problems. Carbonic anhydrase I (CA I), although its exact physiological role is mostly unknown (Gao et al. 2007). Moreover, CA III is connected to oxidative stress and is correlated with several inflammatory conditions, such as myasthenia gravis and rheumatoid arthritis (Du et al. 2009; Barreiro and Hussain 2010). The wide range of functions performed by hCA isoforms makes them crucial targets for therapeutic intervention.

The current study also examines the inhibitory effects of several extracts from *E. californica* on carbonic anhydrase I (hCA I) and II (hCA II) isoenzymes. The examined extracts are presented in Table 4 and consist of Ethyl Acetate (EA), Ethanol, Ethanol/Water, and Water. Acetazolamide is utilized as a standard reference. The results indicate that the EA extract demonstrates the most potent inhibitory effect on hCA I and hCA II, with IC50 values of 2.132 and 2.851 μg/mL, respectively. This is significant since the values are similar to the standard medicine acetazolamide, which has IC50 values of 0.154 μg/mL for hCA I and 0.289 μg/mL for hCA II. This suggests that the EA extract is highly effective (Table 4).

**TABLE 4:** Inhibition of carboxylic anhydrase isoenzymes I and II.

Extracts	IC_50_ (μg/mL)			
	hCA I	*r* ^2^	hCA II	*r* ^2^
EA	2.132	0.9612	2.851	0.9239
Ethanol	86.625	0.9663	138.6	0.9465
Ethanol/Water	99	0.9931	115.5	0.9418
Water	99	0.9758	77	0.9159
Asetazolamid	0.154	0.985	0.289	0.9514

The ethanol extract exhibited moderate inhibitory action, as evidenced by IC50 values of 86.625 μg/mL for hCA I and 138.6 μg/mL for hCA II. Similarly, the ethanol/water extract demonstrated IC50 values of 99 μg/mL for hCA I and 115.5 μg/mL for hCA II, which likewise suggests a moderate activity level. The aqueous extract exhibited moderate activity, with an IC50 value of 99 μg/mL for hCA I and 77 μg/mL for hCA II. Although their IC50 values are larger than those of the EA extract and acetazolamide, they demonstrate potential as natural inhibitors of carbonic anhydrase isoenzymes.

Prior research on *E. californica* has predominantly concentrated on its alkaloid composition, specifically examining substances such as californidine and escholzine, which are known to contribute to its proven pain‐relieving and sleep‐inducing qualities (Hosseini et al. 2024; Sleep 2021). Nevertheless, the present study emphasizes a novel therapeutic capability of this plant as a suppressor of carbonic anhydrase isoenzymes. Carbonic anhydrase inhibitors are crucial in treating a range of medical diseases, including glaucoma, epilepsy, and altitude sickness (Supuran 2021; Er 2023).

The EA extract's significant efficacy indicates its potential as a natural substitute for synthetic carbonic anhydrase inhibitors. Moreover, the modest activity level seen in the other extracts (ethanol, ethanol/water, and water) suggests that various extraction procedures can potentially produce bioactive chemicals from *E. californica* that may have therapeutic implications.

These findings provide opportunities for additional research to identify and describe the chemicals in the EA extract responsible for the observed inhibition of enzymes. In addition, investigating the combined effects of these substances could offer a more comprehensive understanding of how they work and improve their potential for medical treatment.

### 3.9 Cytotoxic Effects

**FIGURE 3:**
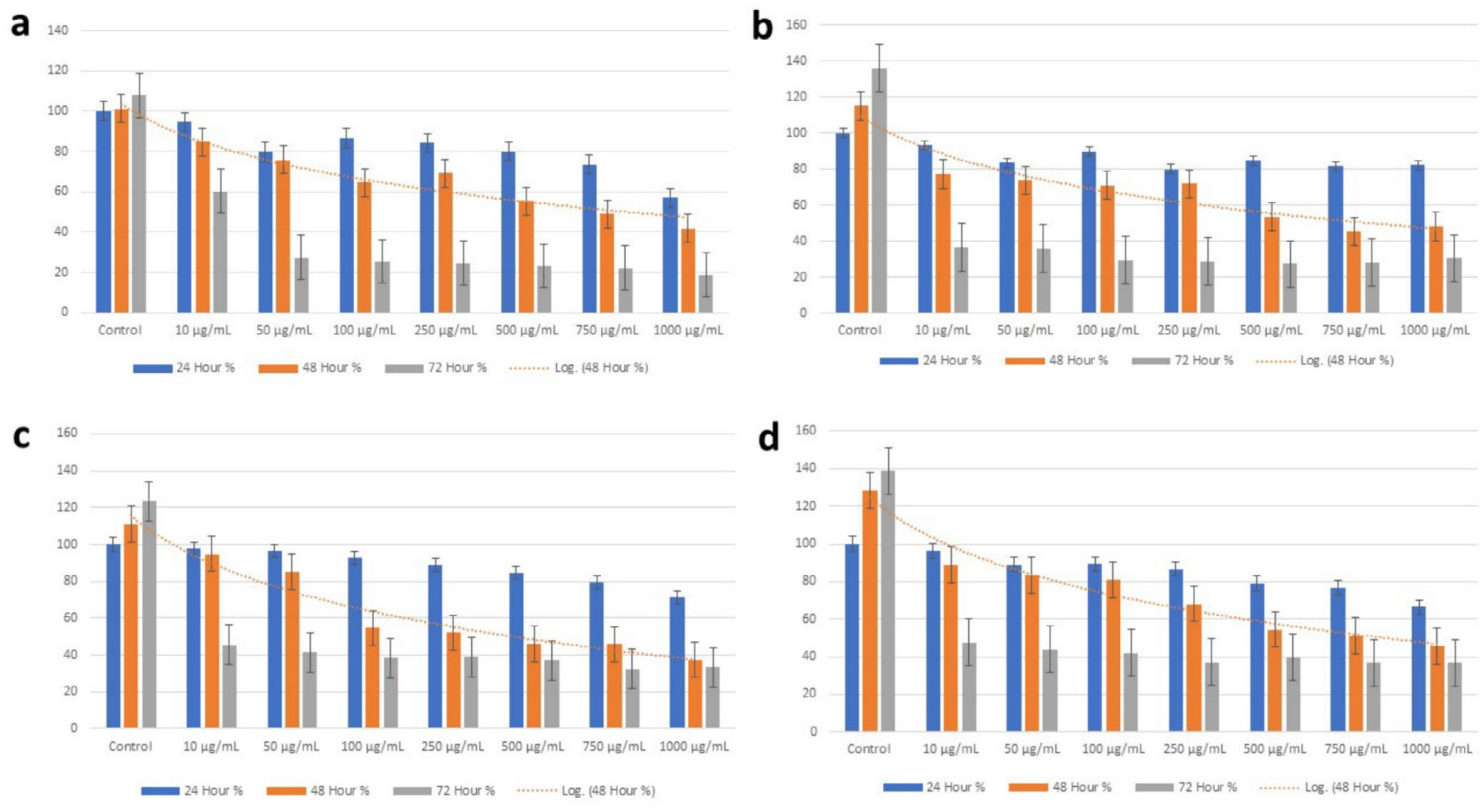
The cytotoxic effect graph of *E. californica* extracts on A549 cancer cell % viability: (a) Ethyl acetate, (b) ethanol, (c) ethanol/water, and (d) water.

The cytotoxic effects of the extracts on A549 lung cancer cells were determined by applying different concentrations of extracts (ethyl acetate, ethanol, ethanol/water, water) at 24, 48, and 72 h intervals and with various doses (0–1000 μg/mL). According to the MTT test results, IC_50_ values were calculated, and the cytotoxic values found for *E. californica* extracts were recorded. The IC_50_ value for ethyl acetate, ethanol, ethanol/water, and water extracts was found at the 48th hour: 750, 500, 250, and 750 μg/mL, respectively. Ethanol and ethanol/water extracts showed a higher cytotoxic effect than the others (Figure 3).

### 3.10 Gene Expression Results

**FIGURE 4:**
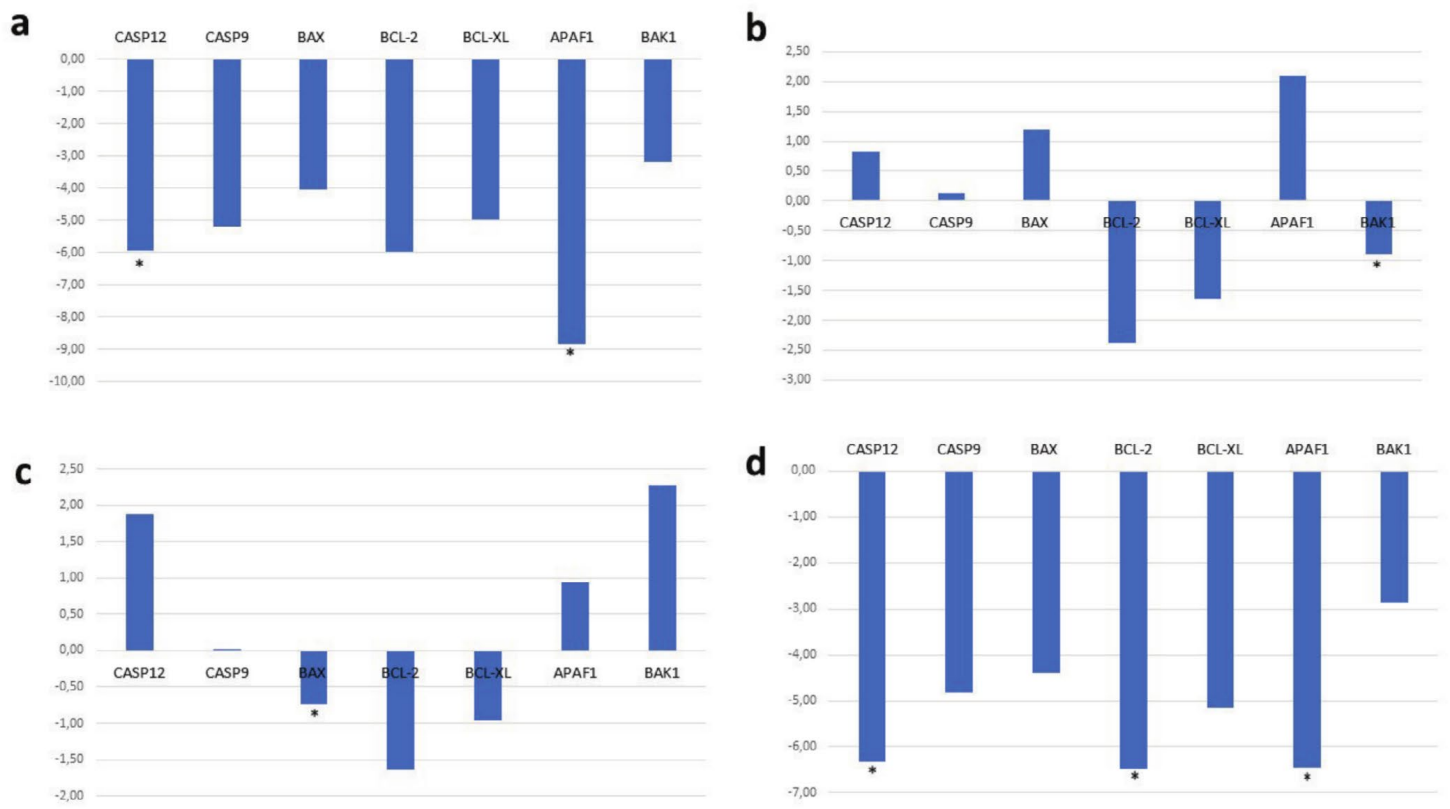
Fold‐change graph of the result of gene expression analyses of apoptosis‐related and caspase pathway genes in A549 cell line: (a) Ethyl acetate, (b) Ethanol, (c) Ethanol/water, (d) Water.

To evaluate the effect of *E. californica* on the apoptosis pathway, the expression levels of apoptosis‐related genes (*Bax*, *Bak1*, *Bcl‐2*, *Bcl‐XL*, *Apaf‐1*, *Casp9*, *Casp12*) were investigated in A549 cells. According to our results, *E. californica* partially/weakly triggers apoptosis in A549 lung cancer cells. In general, ethanol and ethanol/water extracts were more successful in triggering apoptosis than ethyl acetate and water extracts. After ethyl acetate extract was applied to A549 cells, a statistically significant down‐regulation was detected in the expression of all genes examined (*Casp12* and *Apaf1 p* > 0.05). While ethanol extract caused down‐regulation of *Bak1*, *Bcl‐2*, and *Bcl‐XL* genes in A549 cells, it caused up‐regulation in other genes (*Bak1 p* > 0.05). Ethanol/water extract down‐regulated anti‐apoptotic genes (*Bcl‐2*, *Bcl‐XL*) and *Bax* gene in A549 cells (*Bax p* > 0.05), while other pro‐apoptotic genes were up‐regulated. In water extract applied to A549 cells, the expression of all genes was down‐regulated (*Casp12*, *Bcl‐2*, *Apaf1 p* > 0.05) (Figure 4).

**FIGURE 5:**
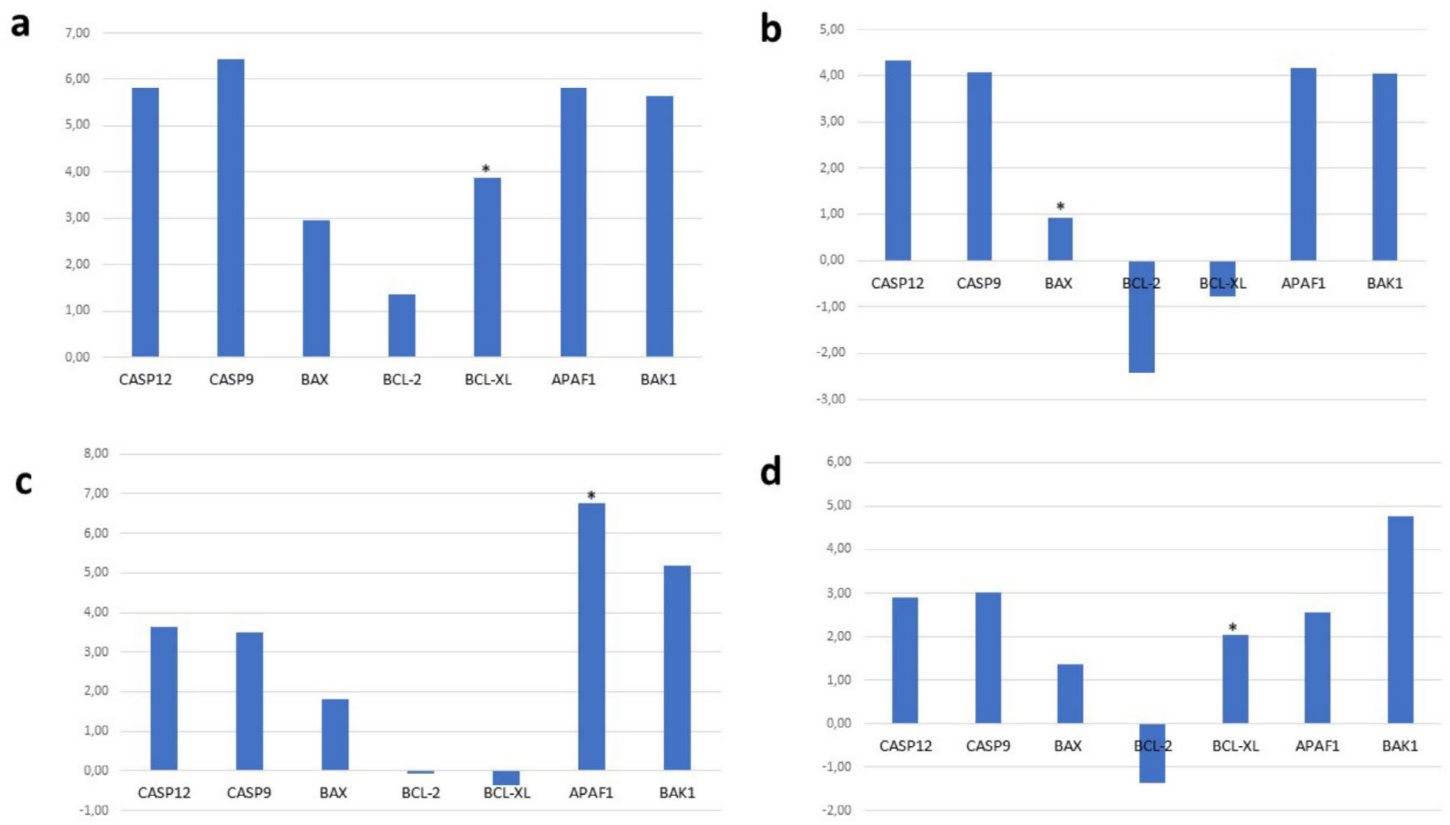
Fold‐change graph of the result of gene expression analyses of apoptosis‐related and caspase pathway genes in HEK293 cell line: (a) Ethyl acetate, (b) Ethanol, (c) Ethanol/water, (d) Water.

According to the studies conducted on the HEK293 cell line, the ethyl acetate extract caused significant up‐regulation in all genes (*Bcl‐XL p* > 0.05). After applying the ethanol and ethanol/water extracts, similar results were obtained by detecting down‐regulation in the expression of only antiapoptotic genes and up‐regulation of other genes. The application of the water extract resulted in down‐regulation in the expression of all genes except the *Bcl‐2* gene (*Bcl‐XL p* > 0.05) (Figure 5).

In fact, according to our gene expression results, the mechanism of triggering the apoptosis pathway against lung cancer for *E. californica* is not clear. However, ethanol and ethanol/water extracts seem to be more successful in driving the cell to apoptosis. In addition, our MTT analysis results determined that these extracts were more cytotoxic and their total phenolic and flavonoid contents were higher than those of other extracts (Figure 3; Table 1).

Usually, down‐regulation of anti‐apoptotic genes that prevent cell death and up‐regulation of pro‐apoptotic proteins trigger the release of cytochrome c in the intrinsic pathway, thus initiating the apoptosis process (Jan 2019; Kashyap et al. 2021). However, *E. californica* did not act as a clear apoptosis inducer in lung cancer cells. This can be explained by *E. californica* preventing or delaying the cell from entering apoptosis. Whether the cell undergoes apoptosis or not depends on the totality of intracellular signals and other factors in the cellular microenvironment (inflammatory signals, toxic substances, etc.) (Apostolova and Victor 2015; Gusev and Zotova 2019; Tkachenko 2024). In addition, the status of other apoptotic and anti‐apoptotic pathways should also be taken into account.

On the other hand, cellular stress can reduce the expression of both antiapoptotic and proapoptotic genes (Fulda et al. 2010; Kannan and Jain 2000; O'Brien and Kirby 2008). Cellular stress, intracellular and extracellular stress factors (oxidative stress, hypoxia, toxins, etc.) can activate apoptotic and antiapoptotic pathways simultaneously (Kannan and Jain 2000; Chandra et al. 2000; Poh Loh et al. 2006). This instability can disrupt cellular homeostasis and cause cytotoxicity. In addition, mitochondrial dysfunction disorders can cause dysregulation of apoptotic signals by disrupting energy production in the cell (Kilbride and Prehn 2013; Soane et al. 2007). Since the increase in *Casp12* expression observed after *E. californica* application is closely related to cellular stress mechanisms such as ER stress (Creagh et al. 2003), it suggests that cellular stress mechanisms are at play here. These findings underscore the potential of *E. californica* in modulating cellular stress pathways, which are of paramount importance in the context of neuropsychiatric disorders and insomnia. It is established that conditions such as schizophrenia, attention deficit hyperactivity disorder (ADHD), depression, and insomnia are associated with dysregulation of apoptosis and cellular stress mechanisms. The observed effects of *E. californica* on apoptosis and cellular stress genes indicate its potential for therapeutic applications in the management of neuropsychiatric disorders and insomnia.

### 3.11 Network Pharmacology

**FIGURE 6:**
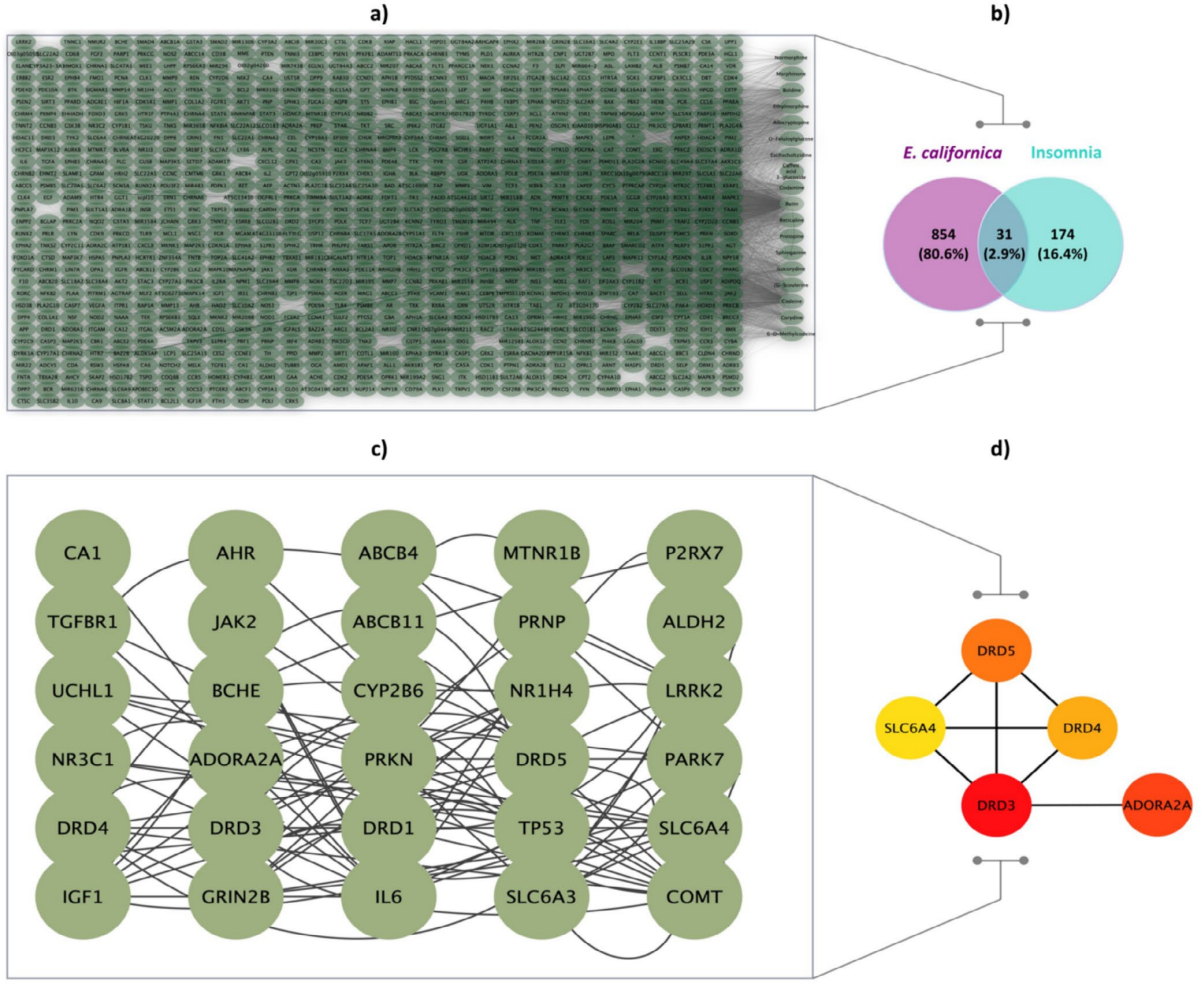
Target analysis of *E. californica* and insomnia. (a) Venn diagram showing the overlap between *E. californica* ‐related genes and insomnia‐associated genes. (b) Interaction between active compounds and target genes involved in insomnia. Insomnia interaction network: (c) Mapping the network of protein targets (d) Hub genes related to insomnia.

**FIGURE 7:**
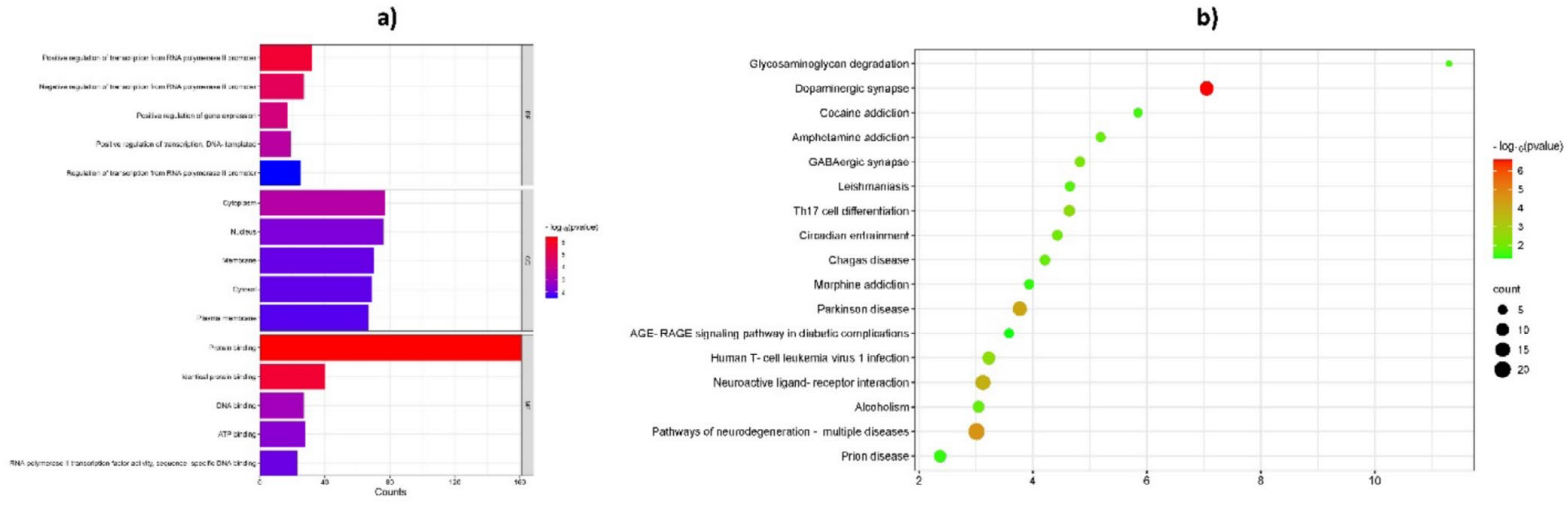
Cancer‐related term enrichment analysis using KEGG and GO. Cellular elements, molecular functions, and biological processes are the three areas that make up the ontology. The Y‐axis shows the enrichment values, and the X‐axis shows the top five GO terms for each domain. The Y‐axis shows the enrichment value, and the X‐axis shows the increasing number of terms for the top 30 most important terms for the KEGG analysis.

A search was conducted in the PubChem, STITCH, SwissTarget, and CTD databases to determine genes associated with the eighteen phytochemicals of *E. californica* . Following the removal of duplicate gene pairs, 885 genes were recognized as potential targets. Among the compounds, those of rutin and codeine are notable for having the highest number of nodes. The network map illustrates the shared molecular targets among the compounds, thereby demonstrating the extensive many‐to‐many relationships between the compounds and their targets. The genes associated with insomnia were screened through STRING, CTD, DisGeNET, and GeneCards platforms. Only genes supported by direct experimental evidence were retained from the CTD database. In the DisGeNET database, gene entries with association scores of 0.010 or below were included. GeneCards data were filtered to retain disease‐associated genes that code for proteins. A total of 205 genes were identified for further investigation. Among the 885 genes associated with *E. californica* phytochemicals, 31 were identified as common. In particular, these 885 target genes correspond to 912 nodes and 2348 edges in the network, which indicates that *E. californica* plays a significant role in therapeutic approaches (Figures 6a and 7b). The Dense Module Network Component (DMNC) strategy was applied via the CytoHubba extension in Cytoscape to pinpoint key genes within the constructed interaction network. The core genes identified were DRD3, ADORA2A, DRD5, DRD4, and SLC6A4 (Figure 6c,d).

### 3.12 GO and KEGG Pathway Enrichment Analysis

GO enrichment analysis was performed to investigate the overlapping genes' functions further. This analysis identified 230 BP, (“positive regulation of gene expression”, negative regulation of transcription from RNA polymerase II promoter, and “positive regulation of transcription from RNA polymerase II promoter”). Notably, no hub genes were found within the BB category. Annotation, the majority of proteins were localized to nuclear and cytoplasmic compartments. Concurrently, the MF was primarily enriched in “protein binding”, which included all hub genes, and “identical protein binding”, which included ADORA2A, SLC6A4, and DRD4. As illustrated in Figure 7, gene functional annotations were classified under GO terms related to biological processes, cellular components, and molecular functions, providing a meaningful understanding of the biological roles and mechanistic relevance of the identified genes within cellular pathways.

Pathway functional pathway analysis was conducted to investigate further the biological roles of intersecting genes, including those potentially related to insomnia. This analysis identified several critical pathways with significant enrichment. The “Pathways of neurodegeneration – multiple diseases” showed considerable enrichment and involved 20 genes, suggesting that neurodegenerative processes do not include the identified hub genes. The “neuroactive ligand‐receptor interaction”, enriched and comprising 16 genes, indicates the importance of neuroactive signaling processes in sleep regulation and insomnia. The identified hub genes in this pathway are ADORA2A, DRD3, DRD4, and DRD5 (Figure 7b).

The “Parkinson disease”, which includes 14 genes, highlights the relevance of neurodegenerative mechanisms to sleep disorders, particularly insomnia, involving ADORA2A. Other notable enriched pathways comprised the “Human T‐cell leukemia virus 1 infection,” “Prion disease”, and “Alcoholism”, involving 10, 9, and 8 genes, respectively. Notably, the “Alcoholism” pathway includes only ADORA2A. Further pathways such as “Th17 cell differentiation”, “GABAergic synapse”, “Circadian entrainment”, and “Chagas disease” were also enriched, each involving 6–7 genes. Pathways related to addiction, such as “Amphetamine addiction”, “Morphine addiction”, and “Cocaine addiction,” were identified with4‐5 genes, highlighting potential targets. Lastly, the “AGE‐RAGE signaling pathway in diabetic complications” and “Glycosaminoglycan degradation” pathways involved 5 and 3 genes respectively, indicating their roles in metabolic and structural processes (Figure 7b). These findings provide a comprehensive overview of the enriched pathways, offering insights into the complex biological networks and potential disease mechanisms associated with the intersecting genes, with a particular focus on their implications for insomnia. The analysis reveals significant pathways and biological processes related to insomnia, emphasizing the role of neuroactive signaling and neurodegenerative processes. Hub genes such as ADORA2A, DRD3, DRD4, and DRD5 are particularly noteworthy due to their involvement in critical pathways. The identified pathways provide potential targets for further research into the mechanisms of insomnia and related disorders.

### 3.13 Molecular Docking Result

**FIGURE 8:**
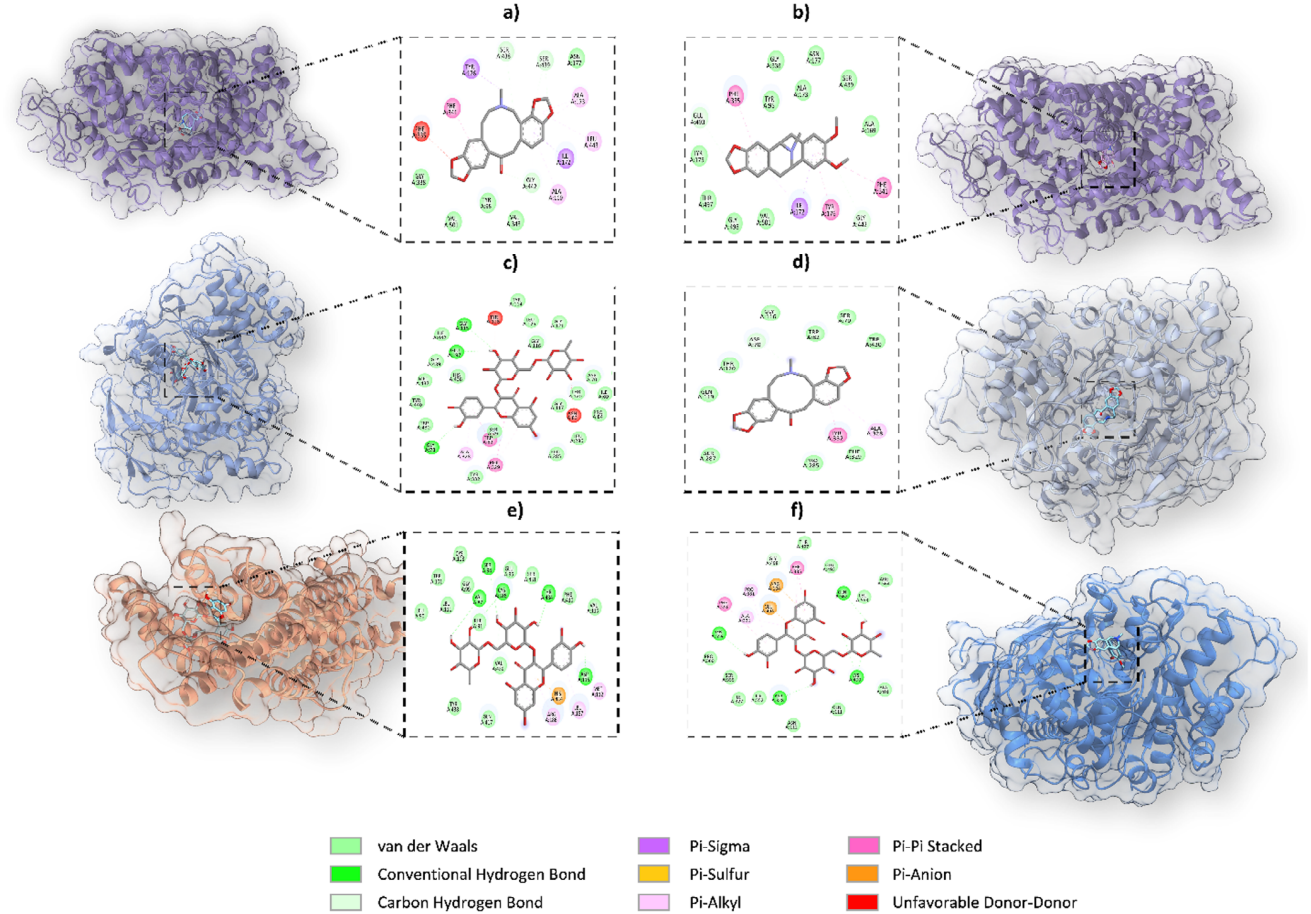
Enzymes and proteins' active sites with compounds showing the best binding energy: (a) Interaction between SERT and protopine; (b) interaction between SERT and eschscholtzidine; (c) interaction between BChE and rutin; (d) interaction between BChE and protopine; (e) interaction between DRD4 and rutin; (f) interaction between SERT and rutin.

Molecular docking procedures were performed on selected proteins relevant to the study. Details of the grid dimensions and coordinates are provided in Table S5. Out of the diverse pool of phytochemicals derived from *E. californica* , eighteen were selected for further investigation based on their widespread interactions. These candidates exhibited binding affinities between −11.1 and −4.2 kcal/mol against insomnia‐related enzymes and proteins (Table S2). Compounds that achieved a docking score ≤ −8.0 and established at least one hydrogen bonding interaction were included in the detailed analysis. Table 6 provides an overview of binding affinities and associated amino acid contacts, with graphical representations provided in Figure 1. Additional results for ligands with scores below −8.0 are listed in Table S2. Among the eighteen molecules evaluated, protopine, rutin, and eschscholtzidine showed particularly strong interactions with the targets. These three compounds displayed significantly favorable docking scores and were found to interact effectively with various proteins and enzymes. A closer look at the binding patterns revealed enhanced affinities for the active sites of proteins such as SERT and DRD4. Non‐covalent interactions such as π–σ, π–sulfur, π–alkyl, and π–π stacking types were frequently observed and, in many cases, occurred more frequently than traditional hydrogen bonds (Figure 8). Notably, 45 of the docked complexes exhibiting binding scores lower than −8.0 kcal/mol lacked hydrogen bonds and are documented in Table S2.

However, hydrogen bond interactions were identified in some notable cases: between DRD5 and rutin (9), BChE and caffeic acid hexoside (6), AChE and rutin, and several others including DRD4 and rutin, and SERT and rutin (5). These results positioned rutin as a key ligand in multiple interactions. Protopine demonstrated the most favorable binding affinity among all tested compounds, yielding a docking score of −11.1 kcal/mol against SERT, despite the lack of hydrogen bond formation. This finding suggests a strong potential for inhibitory activity. Overall, this suggests that both hydrogen bonding and non‐covalent interactions are critical for the binding performance of these phytochemicals (Table 5; Figure 8a).

**TABLE 5:** The docking score (kcal/mol) and interacting residues of the enzyme and protein.

Compound	Receptor	Binding energy (kcal/mol)	Interaction		Amino acid residues
	Target		Type	Number	Binding site
rutin	AChE	−9.7	H‐bond	5	Ser A:293, Phe A:346, Phe A:295, Arg A:296, Tyr A:124
morphinone	AChE	−9.8	H‐bond	2	Ser A:293, Arg A:296
normorphine	AChE	−9.4	H‐bond	1	Glu A:202
scoulerine	AChE	−9.3	H‐bond	1	Tyr A:124
O‐feluloylglucose	AChE	−8.5	H‐bond	5	Trp A:86, Tyr A:124, Gly A:122, Ala A:204thr A:83
caffeic acid hexoside	AChE	−8.8	H‐bond	4	His A:447, Gly A:121, Gly A:122, Tyr A:337
codamine	AChE	−8.9	H‐bond	1	Ser A:293
reticuline	AChE	−8.7	H‐bond	2	Trp A:86, Ala A:204
allocryptopine	AChE	−8.7	H‐bond	2	Tyr A: 124 (2)
rutin	glucosidase	−9.6	H‐bond	4	Asp A:357, Arg A:552, Asn A:475, Lys A:506
normorphine	glucosidase	−8.3	H‐bond	3	Asn A:417, Lys A:421, Phe A:419
protopine	glucosidase	−8.6	H‐bond	1	Asn A:475
rutin	DRD5	−9.2	H‐bond	9	Glu R:102, Asn R:216 (3), Cys R:217 (2), Asp R:218, Val R:336, Asn R:316
morphinone	DRD5	−8.3	H‐bond	1	Ser R:124
methylcodeine	DRD5	−8.0	H‐bond	3	Ser R:124 (2), Trp R:349
normorphine	DRD5	−8.8	H‐bond	2	Ser R:219, Asp R:120
codeine	DRD5	−8.1	H‐bond	3	Ser R:124 (2), Trp R:349
boldine	DRD5	−8.8	H‐bond	3	Ser R:124 (2) Trp R:349
caffeic acid hexoside	DRD5	−8.7	H‐bond	4	Asn R:316 (2), Ile R:121, Gly R:348
reticuline	DRD5	−8.3	H‐bond	1	Lys R:98
rutin	TYR	−8.7	H‐bond	3	Tyr A:362, Asn A:378, Val A:211
protopine	TYR	−8.0	H‐bond	1	His A:392
rutin	A2a	−8.4	H‐bond	2	Ser A:67, Trp A:268
scoulerine	A2a	−8.8	H‐bond	1	Ser A:6
isocorydine	A2a	−9.4	H‐bond	1	Ala A:81
caffeic acid hexoside	A2a	−8.3	H‐bond	1	Ala A:59
codamine	A2a	−8.4	H‐bond	1	Ser A:6
reticuline	A2a	−9.3	H‐bond	1	Ser A:6
rutin	DRD3	−9.0	H‐bond	3	Tyr A:373, Ser A:366, Asn A:352
morphinone	DRD3	−8.3	H‐bond	1	Tyr A:365
normorphine	DRD3	−8.0	H‐bond	1	Tyr A:365
rutin	BChE	−11.0	H‐bond	3	Gly A:215, Glu A:197, Gly A:78
normorphine	BChE	−8.4	H‐bond	1	Asp A:70
scoulerine	BChE	−8.1	H‐bond	1	His A:438
codeine	BChE	−8.5	H‐bond	1	Tyr A:440
corydine	BChE	−8.4	H‐bond	1	Gly A:117
caffeic acid hexoside	BChE	−8.2	H‐bond	6	Trp A:430, Trp A:440, Trp A:82, Pro A:285, Gly A:116, Gly A:117
codamine	BChE	−8.2	H‐bond	1	Glu A:197
reticuline	BChE	−8.6	H‐bond	4	Gly A:117, Gly A:116, Asp A:70, Ser A:70
rutin	DRD4	−10.0	H‐bond	5	Val A:87, Cys A:85, Ser A:94, Thr A:434, Asp A:115
protopine	DRD4	−9.6	H‐bond	2	His A:414, Arg A:186
codamine	DRD4	−8.3	H‐bond	1	Arg A:186
reticuline	DRD4	−8.4	H‐bond	1	Arg A:186
allocryptopine	DRD4	−8.2	H‐bond	1	Csy A:185
rutin	amylase	−9.6	H‐bond	4	His A:305, His A:101, Gln A:63, Asp A:197
normorphine	amylase	−8.0	H‐bond	1	Asp A:197
scoulerine	amylase	−8.4	H‐bond	1	His A:101
boldine	amylase	−8.3	H‐bond	2	Asp A:197, Glu A:133
eschscholtzidine	amylase	−8.4	H‐bond	2	His A:299, His A:305
protopine	amylase	−9.0	H‐bond	1	Gln A:63
allocryptopine	amylase	−8.5	H‐bond	1	Thr A:163
rutin	SERT	−9.9	H‐bond	5	Ser A:559, Gln A:562, Lys A:490 (2), Asp A:328
morphinone	SERT	−8.4	H‐bond	1	Arg A:104
normorphine	SERT	−9.1	H‐bond	1	Tyr A:95
scoulerine	SERT	−9.8	H‐bond	2	Phe A:335, Gly A:442
isocorydine	SERT	−8.8	H‐bond	1	Gln A:332
codeine	SERT	−9.3	H‐bond	1	Ser A:336
corydine	SERT	−8.7	H‐bond	1	Gln A:332
caffeic acid hexoside	SERT	−8.1	H‐bond	2	Gln A:332, Ile A:552
reticuline	SERT	−8.0	H‐bond	1	Arg A:104
rutin	3LXE	−8.5	H‐bond	2	His A:94, Thr A:199
morphinone	3LXE	−8.7	H‐bond	2	His A:200, Thr 199
methylcodeine	3LXE	−8.2	H‐bond	1	Gln A:99
normorphine	3LXE	−8.3	H‐bond	1	Thr A:199
codeine	3LXE	−8.8	H‐bond	2	Thr A:199 (2)
eschscholtzidine	3LXE	−8.2	H‐bond	1	Gln A:92
protopine	3LXE	−9.0	H‐bond	1	Gln A:92
rutin	4IWZ	−8.9	H‐bond	1	Pro A:201, Gln A:92
protopine	4IWZ	−9.0	H‐bond	2	Thr A:200, His A:94

In this study, several amino acid residues were identified as recurring interaction sites for multiple ligands across different target enzymes and proteins (Table 5). For instance, residues Tyr A:124 (rutin, scoulerine, O‐feruloylhexose, allocryptopine) and Ser A:293 (rutin, morphinone, codamine) in AChE; Asp A:197 (rutin, normorphine, boldine) and Gln A:63 (rutin, protopine) in amylase; Gly A:117 (corydine/isocorydine, caffeic acid hexoside, reticuline) and Trp A:440 (caffeic acid hexoside, codeine resuide) in BChE; Ser A:6 (scoulerine, codamine, reticuline) in A2a; Ser R:124 (morphinone, 6‐O‐methylcodeine, codeine resuide, boldine), Trp R:349 (6‐O‐methylcodeine, codeine, boldine), Asn R:316 (rutin, caffeic acid hexoside) in DRD5; Gln A:332 (isocorydine, corydine, caffeic acid hexoside) in SERT; Gln A:199 (rutin, morphinone, normorphine, codeine resuide) in hCA‐I and Arg A:186 (protopine, codamine, reticuline) in DRD4 were frequently participated in molecular interactions. The residues positioned at the active sites were consistently engaged by multiple ligands, underscoring their pivotal role in ligand–receptor interactions. Interestingly, various compounds targeting AChE, BChE, amylase, and TRP were observed to interact with overlapping amino acid residues (Yagi et al. 2024). The recurrence of interactions at these residue positions indicates their strategic significance in the rational design of potent inhibitors and emphasizes their potential utility in therapeutic intervention strategies.

Multiple phytochemicals exhibited significant binding affinities toward key enzymes. Rutin and normorphinone exhibited robust interactions with AChE; rutin and reticuline demonstrated significant interactions with BChE; rutin and protopine exhibited notable interactions with GGs, TYR, and amylase—each combination yielding low binding energy values, indicative of high affinity. A number of compounds—including rutin, morphinone, protopine, eschscholtzidine, and scoulerine—exhibited significant inhibitory potential against AChE, BChE, TYR, GGs, and amylase. These findings support the compounds' relevance in the field of enzyme‐targeted drug discovery. Furthermore, a more extensive group comprising rutin, protopine, eschscholtzidine, scoulerine, methylcodeine, allocryptopine, boldine, ethylmorphine, morphinone, reticuline, and sphinganine exhibited considerable inhibitory activity on neuronal targets such as DRD4, DRD5, SERT, and A2A. Notably, docking simulations highlighted rutin and boldine as potential inhibitors of DRD5; rutin and protopine for DRD4; and rutin and scoulerine for SERT. Collectively, these results underscore the therapeutic promise of these bioactive molecules against protein targets associated with various diseases (Table 5). Furthermore, the data underscore the significance of elucidating the structural underpinnings of ligand–protein interactions to inform the design of novel inhibitors with enhanced efficacy. Such compounds could provide promising leads for developing targeted therapies for diseases associated with DRD5, DRD4, and SERT dysregulation, potentially improving treatment efficacy and patient outcomes.

### 3.14 In Silico Prediction of Pharmacokinetic Parameters

A range of physicochemical parameters and pharmacologically relevant properties was evaluated using the pkCSM platform to predict ADME characteristics. Among the predicted descriptors were aqueous solubility, gastrointestinal absorption, blood–brain barrier (BBB) permeability, and potential hepatotoxicity. The ability of compounds to penetrate the BBB is particularly important in the context of central nervous system (CNS) disorders, as the BBB serves as a tightly regulated interface that restricts the entry of therapeutic agents into brain tissue. This selective permeability poses a major challenge in the treatment of neurological conditions such as Alzheimer's disease, Parkinson's syndrome, multiple sclerosis, and insomnia. The therapeutic success of drugs targeting CNS pathologies is largely dependent on their capacity to efficiently traverse the BBB (Viscusi and Viscusi 2020).

Table 6 shows that molecules other than caffeic acid hexoside, normorphine, O‐feruloylhexose, and rutin have an acceptable blood–brain barrier (BBB) permeability. Furthermore, the presence of hepatotoxic substances, including codamine, codeine residue, escholtzidine, corydine, and isocorydine, was identified. Additionally, good free fraction and intestinal absorption were observed. Due to their hepatotoxic effects, these molecules are predicted to cause liver damage. For instance, codeine has been identified in clinical trials as a cause of breathlessness and liver damage (Viscusi and Viscusi 2020). Concerning water solubility, ethylmorphine (−1.981) and O‐feruloylglucose (−1.823) were found to be highly soluble, whereas (S)‐scoulerine (−2.941), boldine (−3.756), caffeic acid 3‐glucoside (−2.435), codeine (−3.047), corydine (−3.927), escholtzidine (−3.783), isocorydine (−3.839), morphinone (−2.835), normorphine (−2.723), rutin (−2.892), protopine (−3.825) and reticuline (−3.856) were moderately soluble. The poorly soluble category includes 6‐O‐methylcodeine (−5.044), allocryptopine (−4.06), and sphinganine (−5.044). However, it is not desirable for molecules crossing the blood–brain barrier to be highly soluble in water (Mustarichie et al. 2022). Therefore, molecules in the moderately soluble and poorly soluble groups are valuable, i.e., molecules other than ethylmorphine and O‐feruloylhexose. In terms of intestinal absorption, all molecules have good intestinal absorption except caffeic acid hexoside and rutin. In general, the free fraction is acceptable, except for codamine. This classification guides pharmacological research for compounds with favorable ADMET and drug‐like properties, thereby improving the efficiency of drug discovery. Additionally, scoulerine, methylcodeine, allocryptopine, boldine, ethylmorphine, morphinone, protopine, reticuline, and sphinganine can be potential insomnia inhibitors. However, upon examination of the docking scores, it has been determined that boldine, protopine, and scoulerine may potentially inhibit insomnia‐related DRD5, DRD4, and SERT proteins, respectively (Table 6).

**TABLE 6:** Predicted ADME profiles for the compounds analyzed.

S. No	Compound	Water solubility	Intestinal absorption	Fraction unbound	BBB	Hepatotoxicity
1	scoulerine	−2.941	90.075	0.382	0.095	No
2	methylcodeine	−5.044	91.107	0.328	−0.497	No
3	allocryptopine	−4.06	97.744	0.118	−0.536	No
4	boldine	−3.756	91.245	0.155	−0.475	No
5	caffeic acid hexoside	−2.435	7.521	0.64	−1.289	No
6	codamine	−3.868	92.92	0.092	−0.433	Yes
7	codeine resuide	−3.047	96.885	0.365	0.013	Yes
8	corydine	−3.927	94.389	0.121	−0.612	Yes
9	eschscholtzidine	−3.783	96.377	0.138	−0.247	Yes
10	ethylmorphine	−1.981	96.282	0.491	0.093	No
11	isocorydine	−3.839	93.838	0.126	−0.505	Yes
12	morphinone	−2.835	96.409	0.437	−0.046	No
13	normorphine	−2.723	79.133	0.491	−0.14	No
14	O‐feruloylhexose	−1.823	32.123	0.476	−1.496	No
15	protopine	−3.825	97.593	0.134	−0.226	No
16	reticuline	−3.856	91.276	0.134	−0.502	No
17	sphinganine	−5.044	91.107	0.328	−0.497	No
18	rutin	−2.892	23.446	0.187	−1.899	No

*Note:* Good Intestinal Absorption: > 30%, Readily Crosses BBB: BBB > 0.3, Poorly Crosses BBB: BBB > −1, Water solubility, Highly Soluble: LogS > −2, Moderately Soluble: LogS between −4 and −2, Poorly Soluble: LogS < −4.

## 4 Conclusion

This investigation comprehensively evaluated extracts derived from *E. californica* , focusing on their enzyme inhibitory and antioxidant capacities. Various extraction solvents were employed to isolate and characterize the plant's most biologically active extracts and compounds. Notably, ethanol, water (infused), and Ethanol/Water (70%) extracts displayed heightened levels of polyphenols and flavonoids, indicative of enhanced antioxidant capabilities. Additionally, these botanical extracts have shown promise as valuable sources of enzyme inhibitors associated with diverse human ailments, including neurodegenerative diseases, diabetes, and skin‐related issues. These findings suggest avenues for further investigation into their potential utility in developing nutraceutical products from *E. californica* . Furthermore, the investigation identified 885 target genes associated with *E. californica* 's phytochemicals, 31 of which were common to insomnia. In silico studies demonstrated that protopine, rutin, eschscholtzidine, boldine, and scoulerine exhibited notable inhibitory effects on insomnia‐related proteins, specifically DRD5, DRD4, and SERT. Furthermore, these proteins have been linked to neuropsychiatric disorders, including schizophrenia, attention deficit hyperactivity disorder, and depression. These findings indicate the potential pharmacological applications of *E. californica* 's aerial parts as a source for the development of novel phytopharmaceuticals targeting conditions such as insomnia, schizophrenia, ADHD, and depression. All apoptosis‐related pathways should be evaluated together to understand the cytotoxic effect mechanism fully, and detailed analyses should be performed at both the expression and protein levels (Western Blot). In addition, although *E. californica* is a weak anticancer agent for lung cancer, it is a potential anticancer plant because it also has apoptotic effects on normal cells. Still, it should not be used in a direct therapeutic approach.

## Supporting information


**Table S1:**. List of identified compounds in four extracts in negative and positive ionization modes.
**Table S2:** Abundance of compounds in different solvents (*n* = 3).
**Table S3:**. The primer information for qPCR.
**Table S4:** Relevant enzyme target coordinate of the docking box.
**Table S5:** Relevant protein and enzyme target coordinate of the docking box.
**Figure S1:**. Total ion chromatogram of ethyl acetate extract.
**Figure S2:**. Total ion chromatogram of ethanol extract.
**Figure S3:**. Total ion chromatogram of ethanol: water extract.
**Figure S4:**. Total ion chromatogram of water extract.

## Data Availability

Data will be made available on request.
