# Supplementary material for: Comprehensive Metabolomic Profiling and Biological Activity Analysis of Eschscholzia californica Extracts Using LC‐ESI‐QTOF‐MS
Source: Food Sci Nutr. 2025 Sep 2;13(9):e70885. doi: 10.1002/fsn3.70885 (PMC12405063; doi:10.1002/fsn3.70885)
Supplement: Supplementary file 1 — Table S1:. List of identified compounds in four extracts in negative and positive ionization modes. Table S2: Abundance of compounds in different solvents (n = 3). Table S3:. The primer information for qPCR. Table S4: Relevant enzyme target coordinate of the docking box. Table S5: Relevant protein and enzyme target coordinate of the docking box. Figure S1:. Total ion chromatogram of ethyl acetate extract. Figure S2:. Total ion chromatogram of ethanol extract. Figure S3:. Total ion chromatogram of ethanol: water extract. Figure S4:. Total ion chromatogram of water extract. [file FSN3-13-e70885-s001.docx]

**LC-ESI-QTOF-MS metabolomic analysis**

LC-ESI-QTOF-MS metabolomic analysis was performed using an Agilent 1290 Infinity II system coupled with an Agilent 6546 LC/MS QTOF instrument (Agilent, USA). A column of InfinityLab Poroshell 120 EC-C18 (2x150 mm, 2.7 µm) was used to separate compounds from Agilent (USA). The mobile phase consisted of water (A) containing 0.1% formic acid and methanol, and gradient elution mode was applied: 0-4 min, 85% A; 4-7 min, 75%; 7-9 min, 68% A; 9-16 min, 60% A; 16-22 min, 45% A; 22-28 min, 5% A; 28-30 min, 5% A. The flow rate of the mobile phase was set to 0.5 mL min^-1^, and the column was thermostated at 35 °C. An injection volume was 1 µL. The system operated in both positive and negative ionization modes. The QTOF parameters were configured as follows: scan range of 100-1000 m/z; the drying gas temperature at 160 °C; sheath gas flow rate of 12.0 L/min; sheath gas temperature at 400 °C; capillary voltage set to 5.0 kV; nozzle voltage at 2.0 kV; fragmentor set to 140 V; collision energy employed at 10, 20, and 40 eV. MS/MS data were acquired with a scan range of 50-800 m/z, a retention time window of 0.5 min, an isolation window of 1.3 amu, and an acquisition rate of 3 spectra per second. The analysis continuously monitored two reference masses, 112.9855 m/z and 966.0007 m/z, for mass correction.

The raw data files were processed using Mass Hunter Profinder 10.0 software for time alignment and feature extraction. Parameters for time alignment were set as minimal intensity 1000 counts and maximum time shift 0.5 min plus 0.3%. For feature extraction, the parameters were set as m/z range 100-1000, minimal intensity 5000 counts, retention time tolerance 0.25 min, and mass tolerance 20 ppm plus 2 mDa. Total ion chromatograms are presented in Supplementary Materials Fig. S1-S4.

Statistical analyses were conducted utilizing MetaboAnalyst 4.0. The complete dataset from LC-ESI-QTOF-MS/MS analysis underwent unsupervised principal component analysis (PCA) and supervised Partial Least Squares Discriminant Analysis (PLSDA) to gain a comprehensive understanding of the data. Before analysis, normalization by sum was applied to the samples, and the dataset was further processed through mean-centering. Three replicates for each sample group were performed.

**Supplementary materials**

**Table S1** – List of identified compounds in four extracts in negative and positive ionization modes.

| **N** | **Compound** | **RT, min** | **Formula** | **Adduct** | **Theoretical mass** | **Extracted mass** | **m/z** | **Fr. 1** | **Fr.2** | **Fr.3** | **Fr. 4** | **Fr. 5** | **Mass difference, ppm** | **Ref** |
| --- | --- | --- | --- | --- | --- | --- | --- | --- | --- | --- | --- | --- | --- | --- |
| 1 | Asparagine | 1.26 | C4 H8 N2 O3 | [M-H]- | 132.0535 | 132.0535 | 131.0463 | 114.0195 | 70.0298 | 58.0298 | 41.9985 |  | 0.25 | [1] |
| 2 | N-Glycosyl-L-asparagine | 1.27 | C10H18N2O8 | [M-H]- | 294.1063 | 294.1064 | 293.0989 | 185.0560 | 313.0462 | 114.0196 | 70.0291 |  | -0.23 |  |
| 3 | Gluconic acid | 1.31 | C6 H12 O7 | [M-H]- | 196.0563 | 196.0585 | 195.0508 | 160.8427 | 129.0197 | 99,0085 | 75.0087 | 59.0135 | 0.18 |  |
| 4 | N-(1-Deoxy-1-fructosyl)proline | 1.37 | C6 H12 O7 | [M-H]+ | 277.1175 | 277.1163 | 278.1236 | 260.1129 | 242.1024 | 128.0706 | 70.0650 |  | 0.68 |  |
| 5 | Coriose | 1.38 | C7 H14 O7 | [M-H]- | 210.0740 | 210.0742 | 209.0668 | 129.0192 | 85.0295 | 57.0344 |  |  | 0.22 |  |
| 6 | Glyceric acid | 1.38 | C3H6O5 | [M-H]- | 106.0266 | 106.0266 | 105.0193 | 75.0087 | 72.9930 | 56.9981 |  |  | -0.4 | [2] |
| 7 | Monosacharide | 1.43 | C5 H10 O5 | [M-H]- | 150.0528 | 150.0529 | 149.0456 | 131.0359 | 75.0086 | 56.9978 |  |  | -0.22 |  |
| 8 | Malic acid^1^ | 1.53 | C4 H6 O5 | [M-H]- | 134.0215 | 134.0217 | 133,0143 | 115.0040 | 89.0240 | 72.9929 | 71.0137 | 59.0138 | 0.14 | [2] |
| 9 | N-(1-Deoxy-1-fructosyl)valine | 1.65 | C11 H21 N O7 | [M-H]- | 279.1318 | 279.1319 | 280.1393 | 262.1288 | 244.1181 | 216.1231 | 130.0864 | 118.0864 | 0.82 |  |
| 10 | Glutamic acid | 1.91 | C5 H9 N O4 | [M-H]-/[M+H]+ | 129.0426 | 129.0431 | 130.0500 | 84.0433 | 56.0495 | 41.0386 |  |  | 0.72 |  |
| 11 | Citric acid | 1.92 | C6 H10 O8 | [M-H]- | 192.0270 | 192.0272 | 191.9198 | 111,0088 | 87.0087 | 57.0345 |  |  | 0.26 | [2] |
| 12 | Hydroxyadipic acid | 2.24 | C6 H10 O5 | [M-H]- | 144.1042 | 144.0423 | 143.0306 | 117.0548 | 99.0408 | 71.05004 |  |  | 0.15 |  |
| 13 | Homoisocitrate | 2.24 | C7 H10 O7 | [M-H]- | 188.0321 | 188.0322 | 187.0249 | 143.0356 | 125.0247 | 99.0448 |  |  |  | [2] |
| 14 | Trimethoxyphenyl hexoside | 5.02 | C15 H22 O9 | [M-H]- | 346.1264 | 346.1265 | 271.1025 | 183.7857 | 137.0614 | 96.0181 | 55.0185 |  | 0.17 |  |
| 15 | Caffeic acid hexoside | 6.74 | C15 H18 O9 | [M-H]- | 342.0951 | 342.0952 | 341.0883 | 179.0351 | 161.0244 | 133.0296 | 59.0136 |  |  |  |
| 16 | Dihydroferulic acid -O-glucuronide | 6.90 | C16 H20 O10 | [M-H]- | 372.3212 | 372.1056 | 371.0298 | 195.065 | 135.0443 | 59.0932 |  |  | 0.45 |  |
| 17 | Normorphine/ Norcoclaurine | 6.91 | C16 H17 N O3 | [M+H]+ | 271.1208 | 271.1209 | 272.1283 | 255.1017 | 161.0599 | 107.0491 |  |  | 0.33 | [3, 4] |
| 18 | Methylcodeine | 7.38 | C19 H23 N O3 | [M+H]+/[M-H]- | 313.1678 | 313.1682 | 314.1754 | 269.1173 | 107.0492 |  |  |  | 0.73 |  |
| 19 | Scoulerine | 7.53 | C19 H21 N O4 | [M+H]+ | 327.1471 | 327.1476 | 328.1548 | 297.1124 | 265.0861 | 237.0911 | 190.0864 |  | 0.47 | [5, 6] |
| 20 | Boldine/Isoboldine | 7.79 | C19 H21 N O4 | [M+H]+ | 327.1471 | 327.1472 | 328.1543 | 297.1121 | 190.0862 | 173.0597 | 137.0597 | 327.1472 | 0.5 | [5-7] |
| 21 | Morphine derivate | 8.07 | C19H23NO3 | [M+H]+ | 313.1314 | 313.1317 | 314.1388 | 298.074 |  |  |  |  | 0.67 |  |
| 22 | Propylmalate | 8.62 | C7 H12 O5 | [M-H]- | 176.0685 | 176.0685 | 175.0606 | 115.0399 | 85.0660 | 59.0133 |  |  | 0.4 |  |
| 23 | Epicatechin O-glucuronide | 8.67 | C21 H22 O12 | [M+H]+ | 484.1425 | 483.1353 | 484.1019 | 149.1023 | 121.0349 |  |  |  | 1.29 |  |
| 24 | Isorhamnetin 3-(2G-rhamnosylrutinoside)-7-rhamnoside | 8.69 | C40 H52 O24 | [M+H]+ | 960.2540 | 960.2539 | 961.1923 | 626.1796 | 480.1213 | 317.0654 | 85.0282 |  | 0.28 |  |
| 25 | Scoulerine derivate/ nor(iso)corydine | 8.71 | C19 H21 N O6 | [M+H]+ | 327.1471 | 327.1473 | 328.1546 | 313.1310 |  |  |  |  | 0.5 | [5, 7] |
| 26 | Codamine/ Tembetarine isomer | 8.90 | C20H25NO4 | [M+H]+ | 343.1784 | 343.1789 | 344.1858 | 299.1280 | 175.0752 | 137.0595 | 58.0648 |  | 0.46 | [7] |
| 27 | Quinoline alkaloid | 9.00 |  | [M+H]+ |  | 311.116 | 312.1233 | 281.0810 | 251.0701 | 176/0707 |  |  | 0.32 |  |
| 28 | Codeine resuide | 9.02 | C18 H21 N O3 | [M+H]+ | 299.1521 | 299.1523 | 300.1593 |  |  |  |  |  | 0.47 |  |
| 29 | Quinoline alkaloid | 9.03 |  | [M-H]- | 311.1158 | 311.1157 | 310.1087 | 282.1132 | 147.0324 | 107.0504 |  |  | 0.32 |  |
| 30 | Quinoline alkaloid | 9.09 | C19 H23 N O4 | [M+H]+ | 329.1627 | 329.163 | 330.17002 | 213.0910 | 58.0649 |  |  |  | 0.37 |  |
| 31 | (Iso)corydine isomer 1 | 9.18 | C19 H17 N O5 | [M+H]+/[M;H]/ | 341.1627 | 341.1633 | 342.1702 | 311.1281 | 280.1094 | 265.0860 | 190.0863 | 74.0598 | 0.63 | [5-7] |
| 32 | O-Feruloylhexose | 9.34 | C16H20O10 | [M-H]- | 356.1107 | 356.1109 | 355.1047 | 193.0511 | 175.0401 | 160.0166 | 134.0377 | 59.0145 | -0.111 |  |
| 33 | (Iso)corydine isomer 2 | 9.68 | C20 H23 N O4 | [M+H]+ | 341.1627 | 341.1631 | 342.1702 | 311.1281 | 280.1094 | 265.0860 | 190.0863 | 74.0598 | 0.77 | [5-7] |
| 34 | Reticuline | 10.16 | C19 H23 N O4 | [M+H]+ | 329.1627 | 329.1632 | 330.1704 | 192.1020 | 175.0755 | 137.0597 | 115.0543 |  | 0.37 | [5-7] |
| 35 | Morphine derivate /Deoxyreticuline | 10.26 | C19H23NO3 | [M+H]+ | 313.1678 | 313.1678 | 314.1753 | 283.1330 | 175.0754 | 143.0493 | 121.0647 |  | 0.52 | [7] |
| 36 | Alkaloid | 10.56 |  | [M+H]+ |  | 337.095 | 338.1023 | 323.0782 | 187.0630 | 151.0391 |  |  |  |  |
| 37 | Chelailanthifoline | 10.82 | C19H19NO4 | [M+H]+ | 325.1314 | 325.1324 | 326.1393 | 295.0966 | 190.0863 |  |  |  | -0.7 | [5] |
| 38 | Monoacetylmorphine/ Dimethyl-magnoflorine | 10.98 | C19H21NO4 | [M+H]+ | 327.1834 | 327.1839 | 328.1909 | 283.1329 | 121.0648 | 58.0649 |  |  | 0.25 | [8] |
| 39 | Californidine/ Codeine N-oxide | 11.23 | C18 H21 N O4 | [M+H]+ | 337.1314 | 337.1315 | 338.1389 | 322.1076 | 294.1121 |  |  |  | 0.69 |  |
| 40 | Hydroxymethoxy-C-prenylflavanone | 12.03 | C21 H22 O4 | [M+H]+ | 355.1784 | 355.1785 | 356.1853 | 311.1281 | 296.1042 | 237.0906 | 192.1026 | 58.0649 | 0.89 |  |
| 41 | Alkaloid 3/ Magnoflorine | 12.04 |  | [M+H]+ | 341.1263 | 341.1265 | 342.13382 | 324.1233 | 295.0966 | 265.0860 | 190.0860 |  | 0.63 | [7] |
| 42 | Morphine derivate 3/ Quinoline alkaloid | 12.17 | C19H23NO3 | [M+H]+ | 313.1678 | 313.1681 | 314.1753 | 283.1330 | 175.0754 | 143.0493 | 121.0647 |  | 0.25 | [8] |
| 43 | Alkaloid 2 | 12.22 |  | [M+H]+ |  | 341.1638 | 342.1701 | 311.1279 | 280.1094 | 190/0882 |  |  | 0.57 |  |
| 44 | Boldine/Isoboldine | 12.42 | C19 H21 N O4 | [M+H]+ | 327.1471 | 327.1472 | 328.1543 | 297.1121 | 190.0862 | 173.0597 | 137.0597 |  | 0.73 | [5-7] |
| 45 | Protopine | 12.75 | C20 H19 N O5 | [M+H]+ | 353.1263 | 353.1272 | 356.1456 | 338.1389 | 188.0707 | 149.0598 |  |  | 0.95 | [6] |
| 46 | Allocryptopine | 13.20 | C21H23NO5 | [M+H]+ | 369.1576 | 369.1584 | 370.1654 | 206.0812 | 188.0706 |  |  |  | 1.18 | [6] |
| 47 | Kaempferol deoxyhexosyl-hexoside-hexoside | 13.33 | C33 H40 O20 | [M+H]+/[M-H]- | 756.2113 | 756.2113 | 755.2942 | 300.0272 |  |  |  |  | 0.2 |  |
| 48 | Quercetin -deoxyhexoside-hexoside | 14.43 | C27 H30 O16 | [M-H]- | 610.1534 | 610.1536 | 609.1464 | 301.0323 | 272.0248 |  |  |  | 0.42 |  |
| 49 | Quinoline alkaloid | 14.63 | C18 H23 N O5 | [M+H]+ | 355.142 | 355.1421 | 356.1496 | 338.1388 | 204.1018 | 188.0706 |  |  | 0.67 |  |
| 50 | Kaempferol -hexose-deoxyhexose-deoxyhexoside | 15.13 | C33 H40 O19 | [M+H]+/[M-H]- | 740.2164 | 740.2164 | 739.2095 | 284.0329 |  |  |  |  | 0.63 |  |
| 51 | Isorhamnetin-3-o-[alpha-l-rhamnopyranosyl-  (1->4)-alpha-l-rhamnopyranosyl-(1->6)-beta-glucopyranoside] | 15.31 | C34 H42 O20 | [M+H]+/[M-H]- | 770.2269 | 770.2273 | 771.2344 | 626.1796 | 480.1213 | 317.0654 | 85.0282 |  | 0.13 | [9] |
| 52 | Rutin^1^ | 16.56 | C27 H30 O16 | [M+H]+/[M-H]- | 610.1534 | 610.1538 | 465,1028 | 303.0499 | 129.0547 | 85.0284 |  |  | 0.6 | [10] |
| 53 | Eschscholtzidine | 18.05 | C20 H21 N O4 | [M+H]+ | 339.1471 | 339.1477 | 340.1538 | 295.0960 |  |  |  |  | -1.49 | [6, 10] |
| 54 | Morphinone | 18.48 | C17 H17 N O3 | [M-H]- | 283.1208 | 283.121 | 284.1283 | 213.0780 | 181.0654 |  |  |  | 0.02 |  |
| 55 | Quinoline alkaloid | 19.25 | C18 H23 N O5 | [M+H]+ | 355.142 | 355.1422 | 356.1495 | 338.1391 | 309.1120 | 188.0760 |  |  | 0.71 |  |
| 56 | Ethylmorphine/Dehydronorreticuline | 19.30 | C18 H19 N O4 | [M+H]+/[M-H]- | 313.1314 | 313.1316 | 312.1240 | 178.0509 | 148.0530 |  |  |  | 0.51 | [7] |
| 57 | Quercetin dideoxyhexoside | 19.36 | C27 H30 O15 | [M+H]+/[M-H]- | 594.1585 | 594.1585 | 593.1513 | 285.0389 | 255.0294 | 277.0365 |  |  | 0.17 |  |
| 58 | Isorhamnetin deoxyhexosyl-hexoside | 19.87 | C28 H32 O16 | [M+H]+/[M-H]- | 624.169 | 624.1691 | 623.1620 | 315.0512 | 271/0244 |  |  |  | 0.34 |  |
| 59 | Feruloyl-O-methyldopamine | 20.86 | C19 H21 N O5 | [M+H]+ | 343.142 | 343.142 | 344.1494 | 207.0655 | 175.0383 | 147.0440 | 121.0648 |  | 0.35 |  |
| 60 | Flavonoid glycoside | 20.87 |  | [M-H]- |  | 624.2471 | 623.3297 | 578.3267 | 195.6588 | 87.0450 |  |  |  |  |
| 61 | Isorhamnetin^1^ | 24.79 | C16 H12 O7 | [M-H]- | 316.0567 | 316.0584 | 315.0576 | 301.0321 | 272.0398 | 164.0087 | 107.9584 |  | 0.11 |  |
| 62 | Dihydroxy-hexadecanoic acid | 26.73 | C16 H32 O4 | [M+H]+ | 288.2301 | 288.2301 | 287.22255 | 241,2172 |  |  |  |  | -0.8 |  |
| 63 | Sphinganine | 26.78 | C18H39NO2 | [M+H]+ | 273.2668 | 273.2671 | 274.2743 | 88.0755 | 70.0650 | 57.0797 |  |  | 0.8 |  |
| 64 | Phytosphingosine | 26.81 | C18H39NO3 | [M+H]+ | 317.293 | 317.2931 | 318.2007 | 256.2639 | 88.0758 | 70.0650 |  |  | 0.79 |  |
|  | ^1^confirmed by standard |  |  |  |  |  |  |  |  |  |  |  |  |  |

Table S2 – Abundance of compound in different solvents (n=3)

| **N** | **Compound** | **Solvent** | | | | | | | | | | | |
| --- | --- | --- | --- | --- | --- | --- | --- | --- | --- | --- | --- | --- | --- |
|  |  | **Ethyl acetate** | **Ethyl acetate** | **Ethyl acetate** | **Ethanol** | **Ethanol** | **Ethanol** | **Ethanol:water** | **Ethanol:water** | **Ethanol:water** | **Water** | **Water** | **Water** |
| 1 | Asparagine | 31123 | 32827 | 32915 | 297602 | 298037 | 292692 | 433742 | 431326 | 432570 | 362438 | 376089 | 372948 |
| 2 | N-Glycosyl-L-asparagine | 41810 | 40054 | 44122 | 738461 | 727158 | 732436 | 260139 | 250855 | 252619 | 217962 | 218406 | 221410 |
| 3 | Gluconic acid | 571444 | 573236 | 596042 | 841659 | 837168 | 846104 | 3501173 | 3418718 | 3518718 | 2475498 | 2450015 | 2448181 |
| 4 | N-(1-Deoxy-1-fructosyl)proline | 1 | 1 | 1 | 299871 | 290092 | 297401 | 32084 | 31178 | 32291 | 34467 | 33292 | 34843 |
| 5 | Coriose | 393187 | 407298 | 408795 | 527885 | 544582 | 543430 | 1937507 | 1889173 | 1937528 | 1819916 | 1788764 | 1843557 |
| 6 | Glyceric acid | 978067 | 1002015 | 1027604 | 3342804 | 3238184 | 3124811 | 4570389 | 4490926 | 4523500 | 3951209 | 3894176 | 3922923 |
| 7 | Monosacharide | 573886 | 597099 | 606439 | 556487 | 561864 | 556911 | 1231804 | 1196504 | 1248997 | 1354814 | 1325277 | 1335073 |
| 8 | Malic acid^1^ | 1174475 | 1260058 | 1259946 | 7641314 | 7722231 | 7724646 | 1.73E+07 | 1.72E+07 | 1.73E+07 | 1.71E+07 | 1.68E+07 | 1.69E+07 |
| 9 | N-(1-Deoxy-1-fructosyl)valine | 23755 | 27602 | 26977 | 2512145 | 2486459 | 2517141 | 180787 | 183890 | 189338 | 215522 | 214164 | 216761 |
| 10 | Glutamic acid | 3054838 | 3160578 | 3180454 | 3.23E+07 | 3.28E+07 | 3.26E+07 | 3.14E+07 | 3.10E+07 | 3.13E+07 | 2.81E+07 | 2.76E+07 | 2.77E+07 |
| 11 | Citric acid | 635375 | 626559 | 669231 | 185858 | 200234 | 200356 | 9842258 | 9680216 | 9904958 | 1.07E+07 | 1.05E+07 | 1.05E+07 |
| 12 | Hydroxyadipic acid | 106537 | 109972 | 105404 | 1132423 | 1176765 | 1141259 | 2847349 | 2765493 | 2773553 | 3114441 | 3075475 | 3117853 |
| 13 | Homoisocitrate | 119632 | 130744 | 126491 | 2043681 | 2175962 | 2190219 | 5455028 | 5366511 | 5504778 | 6031440 | 5922097 | 6009543 |
| 14 | Trimethoxyphenyl hexoside | 1 | 1 | 1 | 1069175 | 1034857 | 1076539 | 949587 | 930124 | 955830 | 1 | 1 | 1 |
| 15 | Caffeic acid hexoside | 1 | 1 | 1 | 2712221 | 2657084 | 2716841 | 791919 | 786104 | 789356 | 1 | 1 | 1 |
| 16 | Dihydroferulic acid -O-glucuronide | 87484 | 93700 | 91881 | 264456 | 257888 | 267314 | 217086 | 206240 | 223103 | 167342 | 164145 | 171068 |
| 17 | Normorphine/ Norcoclaurine | 99098 | 109627 | 109320 | 1283464 | 1288863 | 1290593 | 774017 | 776807 | 769229 | 615358 | 619610 | 622329 |
| 18 | Methylcodeine | 628583 | 756603 | 750454 | 1,22E+07 | 1,21E+07 | 1,21E+07 | 8280145 | 8246008 | 8350036 | 6609013 | 6619997 | 6643895 |
| 19 | Scoulerine | 9846940 | 1,12E+07 | 1,15E+07 | 1,60E+07 | 1,62E+07 | 1,63E+07 | 8451028 | 8556533 | 8595535 | 6046864 | 6069288 | 6062076 |
| 20 | Boldine/Isoboldine | 1646909 | 1960501 | 1849679 | 7735470 | 6918255 | 7735708 | 3377465 | 3402456 | 3407292 | 2877890 | 2896268 | 2953495 |
| 21 | Morphine derivate | 1454564 | 1592950 | 1590512 | 8367029 | 8398277 | 8414924 | 6780080 | 6843936 | 6919606 | 4436460 | 4408501 | 4425946 |
| 22 | Propylmalate | 227028 | 251495 | 238901 | 1187477 | 1192821 | 1226519 | 1408621 | 1379050 | 1411528 | 1293630 | 1279638 | 1320632 |
| 23 | Epicatechin O-glucuronide | 1379018 | 1435879 | 1417611 | 1021084 | 998765 | 1012338 | 128743 | 131277 | 133765 | 1 | 1 | 1 |
| 24 | Isorhamnetin 3-(2G-rhamnosylrutinoside)-7-rhamnoside | 363396 | 419728 | 412353 | 484090 | 476897 | 485692 | 1 | 1 | 1 | 1 | 1 | 1 |
| 25 | Scoulerine derivate/ nor(iso)corydine | 1 | 1 | 1 | 7181421 | 7141324 | 7235072 | 3546164 | 3556379 | 3616917 | 2947111 | 2961079 | 3007932 |
| 26 | Codamine/ Tembetarine isomer | 1 | 1 | 1 | 5774537 | 5698520 | 5817282 | 4634526 | 4739812 | 4803407 | 3676407 | 3723148 | 3691450 |
| 27 | Quinoline alkaloid | 6383864 | 7106359 | 7225706 | 1,34E+07 | 1,33E+07 | 1,33E+07 | 5723903 | 5790447 | 5725214 | 3296114 | 3275326 | 3325458 |
| 28 | Codeine resuide | 921257 | 1022672 | 1046691 | 1765173 | 1752001 | 1739819 | 898126 | 900814 | 900141 | 748540 | 749398 | 743251 |
| 29 | Quinoline alkaloid | 875621 | 966076 | 946693 | 1697559 | 1707685 | 1709986 | 599131 | 585859 | 607836 | 344210 | 350898 | 359761 |
| 30 | Quinoline alkaloid | 3516779 | 4051616 | 4165105 | 7889203 | 7977627 | 8003421 | 4592969 | 4620630 | 4625559 | 5433129 | 5370342 | 5487006 |
| 31 | (Iso)corydine isomer 1 | 9860478 | 1,12E+07 | 1,13E+07 | 1,61E+07 | 1,60E+07 | 1,64E+07 | 8617677 | 8665234 | 8967812 | 6328677 | 6261374 | 6341243 |
| 32 | O-Feruloylhexose |  |  |  |  |  |  |  |  |  |  |  |  |
| 33 | (Iso)corydine isomer 2 | 9860478 | 1,12E+07 | 1,13E+07 | 1,61E+07 | 1,60E+07 | 1,64E+07 | 8617677 | 8665234 | 8967812 | 6328677 | 6261374 | 6341243 |
| 34 | Reticuline | 1,03E+07 | 1,15E+07 | 1,16E+07 | 1,73E+07 | 1,72E+07 | 1,72E+07 | 9804926 | 1,00E+07 | 9970296 | 7619382 | 7603542 | 7657105 |
| 35 | Morphine derivate /Deoxyreticuline | 1 | 1 | 1 | 2745260 | 2705072 | 2718808 | 1403727 | 1419683 | 1429057 | 1077891 | 1074162 | 1122814 |
| 36 | Alkaloid | 544613 | 641467 | 629247 | 1519573 | 1515943 | 1524194 | 1014471 | 1013778 | 1025721 | 72466 | 75934 | 75487 |
| 37 | Chelailanthifoline | 2,46E+07 | 2,76E+07 | 2,81E+07 | 4,21E+07 | 4,19E+07 | 4,19E+07 | 2,31E+07 | 2,34E+07 | 2,34E+07 | 1,57E+07 | 1,56E+07 | 1,58E+07 |
| 38 | Monoacetylmorphine/ Dimethyl-magnoflorine | 2872880 | 3235192 | 3271222 | 1,90E+07 | 1,88E+07 | 1,88E+07 | 1,23E+07 | 1,24E+07 | 1,23E+07 | 8878994 | 9027517 | 8937991 |
| 39 | Californidine/ Codeine N-oxide | 1 | 1 | 1 | 3668069 | 3625856 | 3613925 | 2237783 | 2245286 | 2303602 | 1365211 | 1332691 | 1344776 |
| 40 | Hydroxymethoxy-C-prenylflavanone | 3282932 | 3781482 | 3832807 | 7875194 | 7825346 | 7644624 | 6094504 | 6126326 | 6131727 | 4205645 | 4156448 | 4207651 |
| 41 | Alkaloid 3/ Magnoflorine | 9596790 | 1,06E+07 | 1,08E+07 | 2593558 | 2556938 | 2521294 | 1706864 | 1708434 | 1725135 | 1 | 1 | 1 |
| 42 | Morphine derivate 3/ Deoxyreticuline/ N,N-Dimethylcoclaurine | 1 | 1 | 1 | 1 | 1 | 1 | 6191727 | 6374564 | 6465580 | 5179776 | 5170800 | 5133929 |
| 43 | Alkaloid 2 | 4,90E+07 | 5,53E+07 | 5,54E+07 | 8,54E+07 | 8,52E+07 | 8,44E+07 | 5,06E+07 | 5,06E+07 | 5,11E+07 | 3,48E+07 | 3,52E+07 | 3,54E+07 |
| 44 | Boldine/Isoboldine | 4695873 | 5334458 | 5370057 | 1,03E+07 | 1,05E+07 | 1,04E+07 | 4999262 | 5180418 | 5276825 | 4114329 | 4192823 | 4196495 |
| 45 | Protopine | 3,68E+07 | 4,11E+07 | 4,14E+07 | 4,24E+07 | 4,23E+07 | 4,18E+07 | 2,98E+07 | 2,97E+07 | 3,01E+07 | 1,83E+07 | 1,84E+07 | 1,85E+07 |
| 46 | Allocryptopine | 7,05E+07 | 7,81E+07 | 7,93E+07 | 6,49E+07 | 6,48E+07 | 6,43E+07 | 5,84E+07 | 5,86E+07 | 5,91E+07 | 4,10E+07 | 4,11E+07 | 4,12E+07 |
| 47 | Kaempferol deoxyhexosyl-hexoside-hexoside | 158276 | 177560 | 183826 | 2079603 | 2079642 | 2102324 | 2946052 | 2654392 | 2584641 | 2419885 | 2362388 | 2416537 |
| 48 | Quercetin -deoxyhexoside-hexoside | 1 | 1 | 1 | 207587 | 203065 | 205801 | 224117 | 217514 | 225440 | 103110 | 104838 | 107404 |
| 49 | Quinoline alkaloid | 1,28E+07 | 1,46E+07 | 1,49E+07 | 3391774 | 3455277 | 3406723 | 1724112 | 1794061 | 1795283 | 1 | 1 | 1 |
| 50 | Kaempferol -hexose-deoxyhexose-deoxyhexoside | 72440 | 77015 | 75059 | 5028810 | 4945222 | 5080784 | 3194351 | 2987843 | 3061979 | 2032886 | 2033212 | 2020842 |
| 51 | Isorhamnetin-3-o-[alpha-l-rhamnopyranosyl-  (1->4)-alpha-l-rhamnopyranosyl-(1->6)-beta-glucopyranoside] | 171046 | 134855 | 137674 | 6015203 | 6050344 | 6087245 | 4344085 | 4241137 | 4423194 | 3023723 | 2888538 | 3124992 |
| 52 | Rutin^1^ | 521064 | 602849 | 622721 | 9618643 | 9449462 | 9436121 | 5670715 | 5707057 | 5693794 | 2883158 | 2832619 | 2838095 |
| 53 | Eschscholtzidine | 2,11E+07 | 2,40E+07 | 2,49E+07 | 3,78E+07 | 3,80E+07 | 3,78E+07 | 2,17E+07 | 2,23E+07 | 2,23E+07 | 1,05E+07 | 1,06E+07 | 1,06E+07 |
| 54 | Morphinone | 2067135 | 2211658 | 2178646 | 836195 | 850567 | 840140 | 237403 | 229194 | 241099 | 60257 | 61331 | 63976 |
| 55 | Quinoline alkaloid | 9924218 | 1,14E+07 | 1,16E+07 | 3949812 | 3898859 | 3954247 | 2302104 | 2343894 | 2349760 | 1 | 1 | 1 |
| 56 | Ethylmorphine/Dehydronorreticuline | 1,76E+07 | 1,97E+07 | 2,04E+07 | 9516686 | 9536425 | 9676186 | 3109956 | 3137824 | 3155289 | 806765 | 814162 | 825012 |
| 57 | Quercetin dideoxyhexoside | 1 | 1 | 1 | 2090281 | 2043910 | 2107817 | 1228768 | 1274810 | 1279588 | 697578 | 690553 | 687625 |
| 58 | Isorhamnetin deoxyhexosyl-hexoside | 828776 | 978027 | 1000007 | 9343004 | 9177020 | 9284063 | 5994336 | 5992547 | 6038983 | 3643535 | 3722779 | 3718582 |
| 59 | Feruloyl-O-methyldopamine | 3185431 | 4287368 | 4400925 | 2393718 | 2077234 | 1996316 | 1050846 | 982888 | 970622 | 249951 | 227060 | 225530 |
| 60 | Flavonoid glycoside | 249957 | 270390 | 262251 | 149940 | 148033 | 154734 | 85949 | 83958 | 86769 | 10915 | 10473 | 10041 |
| 61 | Isorhamnetin^1^ | 38120 | 43815 | 41867 | 275331 | 268722 | 255603 | 219920 | 210548 | 221995 | 119525 | 118410 | 121163 |
| 62 | Dihydroxy-hexadecanoic acid | 137401 | 154681 | 150085 | 86507 | 92774 | 95163 | 79595 | 76975 | 75578 | 43088 | 39715 |  |
| 63 | Sphinganine | 1,42E+07 | 1,66E+07 | 1,67E+07 | 3502527 | 3430048 | 3407232 | 1594678 | 1573211 | 1563803 | 1504198 | 1475147 | 1477216 |
| 64 | Phytosphingosine | 2381287 | 2904107 | 2868951 | 621289 | 670602 | 660941 | 323517 | 326017 | 320769 | 270942 | 263068 | 252575 |

**Table S3** – The primers informations for qPCR

| **Gene** | **Oligonucleotide Sequence (5′-3′)** | **Amplicon size** |
| --- | --- | --- |
| *Bax* | F-CCCGAGAGGTCTTTTTCCGAG | 155 |
|  | R-CCAGCCCATGATGGTTCTGAT |  |
| *Bak1* | F-CATCAACCGACGCTATGACTC | 192 |
|  | R-GTCAGGCCATGCTGGTAGAC |  |
| *Bcl-2* | F-GGTGGGGTCATGTGTGTGG | 89 |
|  | R-CGGTTCAGGTACTCAGTCATCC |  |
| *Bcl-XL* | F-GAGCTGGTGGTTGACTTTCTC | 119 |
|  | R-TCCATCTCCGATTCAGTCCCT |  |
| *Apaf-1* | F-AAGGTGGAGTACCACAGAGG | 116 |
|  | R-TCCATGTATGGTGACCCAT |  |
| *Casp9* | F-CTCAGACCAGAGATTCGCAAAC | 116 |
|  | R-GCATTTCCCCTCAAACTCTCAA |  |
| *Casp12* | F-AACAACCGTAACTGCCAGAGT | 118 |
|  | R-CTGCACCGGCTTTTCCACT |  |
| *GAPDH* | F-GGAGCGAGATCCCTCCAAAAT | 197 |
|  | R-GGCTGTTGTCATACTTCTCAT |  |

**Table S4.** Relevant enzyme target coordinate of the docking box.

|  | **Receptor** | | | **Coordinate** | | **Reference** |
| --- | --- | --- | --- | --- | --- | --- |
| **Target** | | **Gene** | **PDB ID** | **Grid size X, Y, Z** | **X, Y, Z dimensions** |  |
| AChE | |  | 2y2v | 22 Å x 30 Å x 40 Å | 31.062, 20.311, 11.947 | [11] |
| BChE | |  | 3djy | 30 Å x 30 Å x 30 Å | 44.794, -19.63, -25.227 | [11] |
| TYR | |  | 5m8o | 26 Å x 26 Å x 28 Å | -13.194, 5.341, -26.28 | [11] |
| amylase | |  | 2qv4 | 28 Å 28 Å x 24 Å | 14.188, 48.964, 22.886 | [12] |
| Glucosidase | |  | 3w37 | 42 Å 52 Å X 54 Å | 3.091, −8.008, −4.08 | [13, 14] |
| hCA-I | |  | 3lxe | 40 Å x 40 Å x 40 Å | -19.049, 36.885, 44.812 | [15, 16] |
| hCA-II | |  | 4iwz | 40 Å x 40 Å x 40 Å | 14.059, 4.757, 14.338 | [16, 17] |
| A2a | | ADORA2A | 4eiy | 60 Å × 60 Å × 60 Å | -0.471, 8.935, 17.159 | [18] |
| DRD4 | | DRD5 | 5wiu | 50 Å × 50 Å × 50 Å | -15.46, 19.80, -19.02 | [19] |
| SERT | | SLC6A4 | 5i74 | 120 Å × 75 Å × 110 Å | 49.00,181.39,7.65 | [19] |
| DRD5 | | DRD5 | 8irv | 40 Å × 40 Å × 50 Å | 102.64, 113.13, 78.61 | [19] |
| DRD3 | | DRD3 | 3pbl | 30 Å × 30 Å × 30 Å | 0.247, -14.796, 10.257 | [20] |

**Table S5.** Relevant protein and enzyme target coordinate of the docking box.

| **Compound and Receptor** | | **Binding energy (kcal/mol）** | **Receptor** | **Binding energy (kcal/mol)** | **Receptor** | **Binding energy (kcal/mol）** | **Receptor** | **Binding energy (kcal/mol）** |
| --- | --- | --- | --- | --- | --- | --- | --- | --- |
| **Compound** | **PDB ID** |  | **PDB ID** |  | **PDB ID** |  | **PDB ID** |  |
| rutin | 2y2v | -9.7 | 5m8o | -8.7 | 3djy | -11.0 | 2qv4 | -9.6 |
| morphinone | 2y2v | -9.8 | 5m8o | -6.6 | 3djy | -8.9 | 2qv4 | -8.4 |
| methylcodeine | 2y2v | -9.0 | 5m8o | -6.0 | 3djy | -8.3 | 2qv4 | -7.9 |
| Normorphine/norcoclaurine | 2y2v | -9.4 | 5m8o | -6.9 | 3djy | -8.4 | 2qv4 | -8.0 |
| scoulerine | 2y2v | -9.3 | 5m8o | -6.2 | 3djy | -8.1 | 2qv4 | -8.4 |
| corydine/isocorydine | 2y2v | -8.5 | 5m8o | -7.0 | 3djy | -8.5 | 2qv4 | -7.2 |
| codeine | 2y2v | -9.1 | 5m8o | -6.5 | 3djy | -8.5 | 2qv4 | -8.2 |
| corydine/isocorydine | 2y2v | -8.8 | 5m8o | -6.8 | 3djy | -8.4 | 2qv4 | -7.6 |
| O-feluloylhexose | 2y2v | -8.5 | 5m8o | -6.8 | 3djy | -7.1 | 2qv4 | -6.8 |
| sphinganine | 2y2v | -6.9 | 5m8o | -4.8 | 3djy | -5.6 | 2qv4 | -5.6 |
| boldine/isoboldine | 2y2v | -9.1 | 5m8o | -7.4 | 3djy | -9.1 | 2qv4 | -8.3 |
| caffeic acid hexoside | 2y2v | -8.8 | 5m8o | -6.9 | 3djy | -8.2 | 2qv4 | -7.6 |
| eschscholtzidine | 2y2v | -9.8 | 5m8o | -6.6 | 3djy | -9.5 | 2qv4 | -8.4 |
| protopine | 2y2v | -9.4 | 5m8o | -8.0 | 3djy | -9.9 | 2qv4 | -9.0 |
| codamine/tembetarine isomer | 2y2v | -8.9 | 5m8o | -6.1 | 3djy | -8.2 | 2qv4 | -7.7 |
| reticuline | 2y2v | -8.7 | 5m8o | -6.1 | 3djy | -8.6 | 2qv4 | -7.4 |
| allocryptopine | 2y2v | -8.7 | 5m8o | -7.0 | 3djy | -9.0 | 2qv4 | -8.5 |
| ethylmorphine | 2y2v | -9.0 | 5m8o | -6.8 | 3djy | -8.4 | 2qv4 | -8.2 |
| rutin | 3w37 | -9.6 | 4eiy | -8.4 | 5wiu | -10.0 | 5i74 | -9.9 |
| morphinone | 3w37 | -8.3 | 4eiy | -7.9 | 5wiu | -8.0 | 5i74 | -8.4 |
| 6-O-methylcodeine | 3w37 | -7.2 | 4eiy | -7.2 | 5wiu | -7.8 | 5i74 | -9.0 |
| normorphine | 3w37 | -7.9 | 4eiy | -7.3 | 5wiu | -7.8 | 5i74 | -9.1 |
| scoulerine | 3w37 | -7.5 | 4eiy | -8.8 | 5wiu | -8.5 | 5i74 | -9.8 |
| isocorydine | 3w37 | -7.0 | 4eiy | -9.4 | 5wiu | -8.4 | 5i74 | -8.8 |
| codeine | 3w37 | -7.2 | 4eiy | -7.2 | 5wiu | -7.7 | 5i74 | -9.3 |
| corydine | 3w37 | -6.7 | 4eiy | -7.7 | 5wiu | -8.6 | 5i74 | -8.7 |
| O-feluloylglucose | 3w37 | -6.6 | 4eiy | -6.6 | 5wiu | -7.2 | 5i74 | -6.2 |
| sphinganine | 3w37 | -5.1 | 4eiy | -6.6 | 5wiu | -5.8 | 5i74 | -4.2 |
| boldine | 3w37 | -7.0 | 4eiy | -9.5 | 5wiu | -8.1 | 5i74 | -9.0 |
| caffeic acid hexoside | 3w37 | -7.6 | 4eiy | -8.3 | 5wiu | -7.6 | 5i74 | -8.1 |
| eschscholtzidine | 3w37 | -7.3 | 4eiy | -7.6 | 5wiu | -8.3 | 5i74 | -10.1 |
| protopine | 3w37 | -8.6 | 4eiy | -9.7 | 5wiu | -9.6 | 5i74 | -11.1 |
| codamine | 3w37 | -6.5 | 4eiy | -8.4 | 5wiu | -8.3 | 5i74 | -6.7 |
| reticuline | 3w37 | -7.3 | 4eiy | -9.3 | 5wiu | -8.4 | 5i74 | -8.0 |
| allocryptopine | 3w37 | -7.9 | 4eiy | -9.0 | 5wiu | -8.2 | 5i74 | -9.5 |
| ethylmorphine | 3w37 | -7 | 4eiy | -7.6 | 5wiu | -7.9 | 5i74 | -9.2 |
| rutin | 8irw | -9.2 | 3pbl | -9.0 | 3lxe | -8.5 | 4iwz | -8.9 |
| morphinone | 8irw | -8.3 | 3pbl | -8.3 | 3lxe | -8.7 | 4iwz | -7.5 |
| 6-O-methylcodeine | 8irw | -8.0 | 3pbl | -8.1 | 3lxe | -8.2 | 4iwz | -7.1 |
| normorphine | 8irw | -8.8 | 3pbl | -8.0 | 3lxe | -8.3 | 4iwz | -7.1 |
| scoulerine | 8irw | -7.9 | 3pbl | -8.4 | 3lxe | -7.6 | 4iwz | -7.9 |
| corydine/isocorydine | 8irw | -8.4 | 3pbl | -7.4 | 3lxe | -7.6 | 4iwz | -7.5 |
| codeine | 8irw | -8.1 | 3pbl | -8.2 | 3lxe | -8.8 | 4iwz | -6.8 |
| corydine/isocorydine | 8irw | -7.5 | 3pbl | -7.2 | 3lxe | -7.9 | 4iwz | -7.9 |
| O-feluloylhexose | 8irw | -6.9 | 3pbl | -7.1 | 3lxe | -7.2 | 4iwz | -7.3 |
| sphinganine | 8irw | -6.1 | 3pbl | -6.2 | 3lxe | -5.7 | 4iwz | -5 |
| boldine/isoboldine | 8irw | -8.8 | 3pbl | -7.7 | 3lxe | -7.8 | 4iwz | -7.1 |
| caffeic acid hexoside | 8irw | -8.7 | 3pbl | -7.7 | 3lxe | -7.0 | 4iwz | -7.5 |
| eschscholtzidine | 8irw | -7.2 | 3pbl | -8.5 | 3lxe | -8.2 | 4iwz | -7.5 |
| protopine | 8irw | -8.0 | 3pbl | -9.5 | 3lxe | -9.0 | 4iwz | -9.0 |
| codamine/tembetarine | 8irw | -8.3 | 3pbl | -7.6 | 3lxe | -7.7 | 4iwz | -7.2 |
| reticuline | 8irw | -8.3 | 3pbl | -7.5 | 3lxe | -7.7 | 4iwz | -7.0 |
| allocryptopine | 8irw | -7.4 | 3pbl | -8.9 | 3lxe | -7.9 | 4iwz | -7.7 |
| ethylmorphine | 8irw | -8.1 | 3pbl | -8.2 | 3lxe | -8.3 | 4iwz | -6.9 |

**Fig S1.** Total ion chromatogram of ethyl acetate extract.

**Fig S2.** Total ion chromatogram of ethanol extract.

**Fig S3.** Total ion chromatogram of ethanol: water extract.

**Fig S4.** Total ion chromatogram of water extract

**References**

1. Winkler A, Hartner F, Kutchan TM, Glieder A, Macheroux P. Biochemical evidence that berberine bridge enzyme belongs to a novel family of flavoproteins containing a bi-covalently attached FAD cofactor. J Biol Chem. 2006;281(30):21276-85. Epub 20060525. doi: 10.1074/jbc.M603267200. PubMed PMID: 16728404.

2. Schiller M, von der Heydt H, März F, Schmidt PC. Quantification of sugars and organic acids in hygroscopic pharmaceutical herbal dry extracts. Journal of Chromatography A. 2002;968(1-2):101-11.

3. Sato F. Plant alkaloid engineering. Comprehensive natural products III. 2020:700-55.

4. Bhakuni DS, Singh AN, Tewari S, Kapil RS. Biosynthesis of reticuline. Journal of the Chemical Society, Perkin Transactions 1. 1977;(14):1662-6.

5. Becker A, Yamada Y, Sato F. California poppy (Eschscholzia californica), the Papaveraceae golden girl model organism for evodevo and specialized metabolism. Frontiers in plant science. 2023;14:1084358.

6. Fedurco M, Gregorová J, Šebrlová K, Kantorová J, Peš O, Baur R, et al. Modulatory effects of Eschscholzia californica alkaloids on recombinant GABAA receptors. Biochemistry research international. 2015;2015(1):617620.

7. Macedo AL, Boaretto AG, Silva ANd, Maia DS, Siqueira JMd, Silva DB, Carollo CA. Evaluation of the Effect of Brazilian Savanna (Cerrado) Seasons in Flavonoids and Alkaloids Accumulation: The Case of Duguetia furfuracea. Journal of the Brazilian Chemical Society. 2021;32(9):1840-50.

8. Tian Y, Zhang C, Guo M. Comparative study on alkaloids and their anti-proliferative activities from three Zanthoxylum species. BMC complementary and alternative medicine. 2017;17:1-16.

9. Beck M-A, Häberlein H. Flavonol glycosides from Eschscholtzia californica. Phytochemistry. 1999;50(2):329-32.

10. Mattoli L, Cangi F, Ghiara C, Burico M, Maidecchi A, Bianchi E, et al. A metabolite fingerprinting for the characterization of commercial botanical dietary supplements. Metabolomics. 2011;7:437-45.

11. Jończyk J, Kukułowicz J, Łątka K, Malawska B, Jung Y-S, Musilek K, Bajda M. Molecular modeling studies on the multistep reactivation process of organophosphate-inhibited acetylcholinesterase and butyrylcholinesterase. Biomolecules. 2021;11(2):169.

12. Safithri M. POTENTIAL OF RED BETEL LEAVES (Piper crocatum) AS TYROSINASE INHIBITOR IN SILICO AND IN VITRO. Indonesian Journal of Pharmaceutical Science and Technology. 2024;11(1):45-54.

13. Basnet S, Ghimire MP, Lamichhane TR, Adhikari R, Adhikari A. Identification of potential human pancreatic α-amylase inhibitors from natural products by molecular docking, MM/GBSA calculations, MD simulations, and ADMET analysis. Plos one. 2023;18(3):e0275765.

14. Patil SM, Martiz RM, Satish A, Shbeer AM, Ageel M, Al-Ghorbani M, et al. Discovery of novel coumarin derivatives as potential dual inhibitors against α-glucosidase and α-amylase for the management of post-prandial hyperglycemia via molecular modelling approaches. Molecules. 2022;27(12):3888.

15. Duran T, Peron G, Zancato M, Zengin G, Cetiz MV, Bouyahya A, et al. Harnessing the chemical composition and anti-oxidant, anti-enzymatic, and anti-cancer activities of two Corydalis species (C. erdelii and C. solida) by using in vitro and in silico analysis. Food Bioscience. 2024:104762.

16. Yu J, Zhou Y, Tanaka I, Yao M. Roll: a new algorithm for the detection of protein pockets and cavities with a rolling probe sphere. Bioinformatics. 2010;26(1):46-52.

17. Yagi S, Zengin G, Eldahshan OA, Singab ANB, Selvi S, Cetiz MV, et al. Functional constituents of Colchicum lingulatum Boiss. & Spruner subsp. rigescens K. Perss. extracts and their biological activities with different perspectives. Food Bioscience. 2024:104496.

18. Salamah NN, Aryati WD, Yanuar A. Virtual screening of Indonesian herbal database as adenosine A2A antagonist using AutoDock and AutoDock vina. Pharmacognosy Journal. 2019;11(6).

19. Tian W, Chen C, Lei X, Zhao J, Liang J. CASTp 3.0: computed atlas of surface topography of proteins. Nucleic acids research. 2018;46(W1):W363-W7.

20. Jin H, Wu C, Su R, Sun T, Li X, Guo C. Identifying dopamine D3 receptor ligands through virtual screening and exploring the binding modes of hit compounds. Molecules. 2023;28(2):527.
